# Systematic Evaluation of Rheumatoid Arthritis Risk by Integrating Lifestyle Factors and Genetic Risk Scores

**DOI:** 10.3389/fimmu.2022.901223

**Published:** 2022-07-06

**Authors:** Xing-Hao Yu, Lin Bo, Rong-Rong Cao, Yi-Qun Yang, Pei He, Shu-Feng Lei, Fei-Yan Deng

**Affiliations:** ^1^ Center for Genetic Epidemiology and Genomics, School of Public Health, Medical College of Soochow University, Suzhou, China; ^2^ Jiangsu Key Laboratory of Preventive and Translational Medicine for Geriatric Diseases, Soochow University, Suzhou, China; ^3^ Department of Rheumatology, The Second Affiliated Hospital of Soochow University, Suzhou, China

**Keywords:** rheumatoid arthritis, healthy lifestyle, genetic factor, polygenic risk score, epidemiology

## Abstract

**Background:**

Effective identification of high-risk rheumatoid arthritis (RA) individuals is still a challenge. Whether the combined effects of multiple previously reported genetic loci together with lifestyle factors can improve the prediction of RA risk remains unclear.

**Methods:**

Based on previously reported results and a large-scale Biobank dataset, we constructed a polygenic risk score (PRS) for RA to evaluate the combined effects of the previously identified genetic loci in both case-control and prospective cohorts. We then evaluated the relationships between several lifestyles and RA risk and determined healthy lifestyles. Then, the joint effects of healthy lifestyles and genetic risk on RA risk were evaluated.

**Results:**

We found a positive association between PRS and RA risk (OR = 1.407, 95% confidence interval (CI) = 1.354~1.463; HR = 1.316, 95% CI = 1.257~1.377). Compared with the low genetic risk group, the group with intermediate or high genetic risk had a higher risk (OR = 1.347, 95% CI = 1.213~1.496; HR = 1.246, 95% CI = 1.108~1.400) (OR = 2.169, 95% CI = 1.946~2.417; HR = 1.762, 95% CI = 1.557~1.995). After adjusting for covariates, we found protective effects of three lifestyles (no current smoking, regular physical activity, and moderate body mass index) on RA risk and defined them as healthy lifestyles. Compared with the individuals with low genetic risks and favorable lifestyles, those with high genetic risks and unfavorable lifestyles had as high as OR of 4.637 (95%CI = 3.767~5.708) and HR of 3.532 (95%CI = 2.799~4.458).

**Conclusions:**

In conclusion, the integration of PRS and lifestyles can improve the prediction of RA risk. High RA risk can be alleviated by adopting healthy lifestyles but aggravated by adopting unfavorable lifestyles.

## Introduction

As an autoimmune and inflammatory disease, rheumatoid arthritis (RA) is characterized by persistent synovitis and systemic inflammation that is induced by the attack of the misguided immune system on healthy cells (1). Clinically, RA patients show painful swelling of the joints, joint deformity, and at later stage damage to a wide variety of body systems, including organs such as the heart and lungs (2). Epidemiological studies have shown that the average global prevalence of RA is estimated to be 0.5-1.0% (3, 4).

The pathogenesis of RA is very complex. Genetic factors, environmental factors, and their interaction determine the susceptibility to RA. The heritability of RA is estimated to be 50% to 60% (4), and family history has approximately three to five times the increased risk of developing RA (3). Previous large-scale genome-wide association study (GWAS) analyses have identified a long list of genetic loci associated with RA risk (5). These loci individually have relatively small or moderate effects on susceptibility to RA risk, but whether the combined effects of multiple genetic loci together can be used as an indicator of genetic risk in effectively predicting RA risk is still largely unknown.

The development of RA is also closely related to environmental factors, and we summarize previous studies focused on the association between lifestyle and RA in **Table S1**. An unhealthy daily lifestyle has been shown to increase the risk of developing RA. Dietary patterns play a central role in the risk and progression of inflammatory diseases (6). For example, excessive consumption of red meat promotes inflammation and proinflammatory effects (e.g., excessive intake of red meat, salt, and calories), whereas regular intake of fish rich in oils and fats and consumption of fruit reduces inflammation (7–9). Numerous epidemiological studies have demonstrated that smoking behavior greatly increases the risk of RA. *In vivo* experiments and animal models discovered crucial roles of smoking in the immune system, synovial fibroblasts, and drug response in RA, which were proven to participate in RA development (10). Subjects with regular physical activity had a lower prevalence of RA than those who were less physically active and physically active RA patients had a milder disease course (11). As a crucial role in the development of diseases, obesity shares common inflammatory links with RA (9).

The polygenic risk score (PRS) is an effective tool to assess the genetic risk of diseases by combining the effects of multiple genetic loci and has been widely used for the risk assessment of cancers and psychiatric diseases (12, 13). Several PRS models have also been constructed to predict RA onset previously, and we summarized most of them in **Table S2** (14–16). However, these models have some endogenous limitations (e.g., limited sample size, either in case-control samples or in prospective cohorts; no systematic evaluation of PRS prediction models under different significance levels; the interaction between PRS and environmental factors was not assessed) that lead to limited risk prediction capacity. More importantly, these models have not taken into account environmental factors, although they are important in determining RA alone or by interacting with genetic loci. A study demonstrated the interactions between multiple environmental exposures and RA risk genes (17). However, no study has focused on RA risk prediction by integrating lifestyle and genetic factors.

The UK Biobank is a large prospective cohort study of 500,000 people covering a wealth of information on demographics, risk factors, environmental exposures, disease diagnosis data, and more (18, 19). Based on baseline, follow-up, and genotype data, we performed a systematic study for RA risk prediction. First, we constructed a PRS for RA and further analyzed the combined effects of the previously identified genetic loci on RA risk and their predictive power. We then assessed the relationship between lifestyles and RA risk and classified individuals according to their lifestyles. Finally, we explored the joint effects by integrating genetic effects and lifestyles on predicting RA risk in a large case-control sample and prospective cohort.

## Materials and Methods

### Study Design

We randomly divided the total population into two parts: training (20%) and test sets (80%), and the seed for sampling was set to “1234” and all variables were balanced in the two divided data sets. The appropriate PRS was selected in the training set, and we evaluated associations between selected PRS and RA risk in the test set. Next, we analyzed the associations between RA risk and multiple lifestyle factors and defined them as healthy lifestyles to explore the joint or interactive effects of genetic and lifestyle factors on RA risk in both case-control and prospective cohort analyses.

### UK Biobank Data Sets

The UK Biobank is a large-scale biomedical database containing in-depth genetic and health information from half a million UK participants (http://www.ukbiobank.ac.uk/) (19). The project began in 2006 with the recruitment of participants aged 40-69 years from 22 assessment centers at baseline, with each eligible participant completing a written informed consent form. The project provided detailed health-related information (including nutrition, lifestyle, medications, etc.) through extensive baseline questionnaires, interviews, physical measurements, blood biochemical analyses, and genotyping of all subjects. This provides an unprecedented opportunity to explore the respective contributions of genetic and environmental factors to disease development.

**Figure 1:**
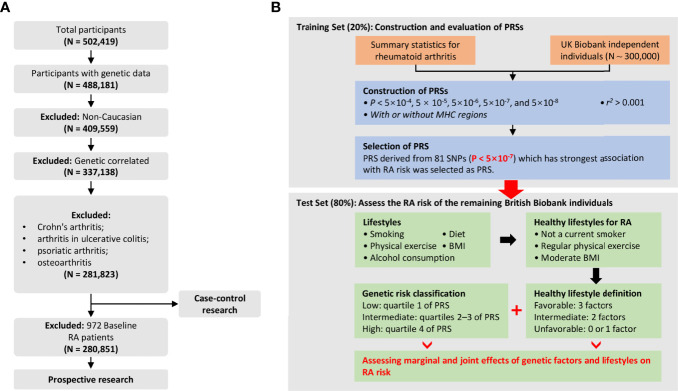
**(A)** Flowchart for filtering participants from the UK Biobank cohort; **(B)** Study design and workflow of our research.

RA was defined by individual medical records obtained from the International Classification of Diseases, Tenth Revision (ICD-10-CM) M05 and M06 in the UK Biobank, which has been commonly used in previous studies (20, 21). Outcomes in the UKB cohort were determined by the onset of RA from baseline up to the date of diagnosis, death, loss to follow-up, or June 31, 2021, whichever occurred first. Therefore, all diagnosed RA patients at baseline were excluded from the prospective study. For individuals, we retained individuals with successfully measured genotypes as well as individuals with white British ancestry. Notably, participants diagnosed with Crohn’s arthritis, arthritis in ulcerative colitis, psoriatic arthritis, and osteoarthritis were excluded from our analysis. The quality control process of the participants is presented in **Figure 1A**. Related field IDs for covariates and ICD-10 codes used for RA diagnosis in the UK Biobank dataset are summarized in **Table S3**.

### Genotyping and Imputation

The genotyping process and quality control process used in the UKB study have been described elsewhere (19). For genotypes, autosomal analysis was restricted to high-quality Haplotype Reference Consortium imputed variants with an MAF > 0.02, imputation information score > 0.5, missing ratio > 0.95, and Hardy-Weinberg *P* > 1×10^−7^. For individuals, we excluded unsuccessful genotyping individuals, nonwhite British individuals, and individuals with at least one relative identified. Finally, up to ~330,000 independent European ancestry individuals with ~9,400,000 high-quality single nucleotide polymorphisms (SNPs) remained for subsequent GWAS analysis.

### Definition of Healthy Lifestyles

According to a review of previous studies (22–25), we considered five healthy lifestyle factors in our analysis, including no current smoking, alcohol consumption, regular physical activity, moderate body mass index (BMI), and healthy dietary pattern: 1. Smoking: Former smokers who had never smoked or had quit for at least 30 years were defined as “nonsmokers”, and others were defined as “smokers”. 2. Alcohol consumption: “Alcohol consumption” was defined as those who drank more frequently than once per month and those whose drinking status was current drinking; “No Alcohol consumption” was defined as no alcohol intake/any alcohol. 3. Physical activity: “Regular physical activity” is defined as those who engage in vigorous activity for at least 75 minutes per week or moderate activity for 150 minutes per week (or an equivalent combination) or vigorous activity and moderate physical activity at least once a week for at least 5 days; others were defined as “No regular physical activity” 4. Diet: “Healthy diet” was defined as those who consumed more fruits, vegetables, whole grains, and fish, as well as less red meat and processed meat; others were defined as an “unhealthy diet”. 5. BMI: “Moderate BMI” is someone whose BMI ranges from 18.5 to 24. Details about the data fields and definitions of healthy lifestyles are summarized in **Table S4**, and all information was collected at recruitment. We included three healthy lifestyle indicators (i.e., no smoking, regular physical activity, and moderate BMI): Include 0 to 1 healthy lifestyle indicator for unfavorable lifestyle, 2 for moderate lifestyle, and all 3 for favorable lifestyle.

### Construction of Polygenic Risk Score

RA-associated SNPs and corresponding summary statistics were accessed from the largest European RA GWAS analysis (5). The genotype of each SNP was obtained from the UK Biobank. RA-associated SNPs were selected at a conservative threshold by using the clumping procedure of PLINK (26): linkage disequilibrium (LD) clumping was performed to identify independent IVs with 1000 Genomes Projects as a reference panel, where the linkage disequilibrium *r* ^2^ was set to 0.001 and the window was set to a 1 MB physical distance. SNPs associated with RA were selected under different cut-offs (P-value < 5×10^-4^, 5×10^-5^, 5×10^-6^, 5×10^-7^, 5×10^-8^), containing 674, 208, 117, 80, and 58 SNPs, respectively. Then, PRSs for RA can be calculated by ${\text{PRS}}_{i}=\sum_{k=1}^{K}X_{ik}\hat{\beta },$ where ${\hat{\beta }}_{k}$ is the SNP estimated effect for the *k* ^th^ SNP, and *X_ik_* is the genotype (0, 1, 2) of the *k* ^th^ SNP index for the *i* ^th^ individual. Finally, a total of 5 PRSs were generated under 5 different thresholds.

To validate the PRSs, the dataset was randomly divided into a training set (20%, N = 56,364) and a test set (80%, N _case-control_ = 225,459, N _prospective_ = 224,927). Each PRS was standardized with a mean zero and standard deviation of one over the entire dataset. Next, we employed logistic regression to model the associations between the 5 PRSs and RA risk, adjusting for fifteen covariates [i.e., age, sex, Townsend deprivation index (TDI) (27), genotyping chip (UKB vs BiLEVE), and top 10 genetic principal components (PCs)]. The best-performing PRS, in terms of highest association and area under the curve (AUC), was selected as the final PRS and held fixed for further analysis in the test set. The genetic risk groups were divided by PRS: low genetic risk (bottom quartile of PRS), intermediate genetic risk (quartiles 2 to 3), and high genetic risk (top quartile). Additionally, considering the complex genetic structure of SNPs, we derived the best-performing PRS after removing SNPs encoded by genes within the human major histocompatibility complex (MHC) region (chr6: 28,477,797 ~ 33,448,354) (PRS _non-MHC_), containing a total of 68 SNPs.

### Statistical Analysis

Independent samples t-test and Manny Whitney U-test were used to compare PRS between RA patients and controls. Multivariable regression models were used to evaluate the association after adjusting for potential confounders (i.e., age, sex, TDI, genotyped batch, assessment center, top 10 PCs). Specifically, multivariable logistic regression was used to assess associations between genetic and lifestyle factors and RA incidence, and odds ratios (ORs) with 95% confidence intervals (CIs) were calculated to measure RA risk in a case-control study. In a prospective study, multivariable Cox regression was utilized, and hazard ratios (HRs) with 95% CIs were calculated to measure RA risk over time. The prediction accuracy of the PRS model was assessed using the AUC with the “pROC” package (28), and a larger AUC indicated better prediction accuracy of the model. Cumulative incidence rates were evaluated within different subgroups in a prospective study. The additive interaction analysis between genetic risk and lifestyles was assessed by relative excess risk for interaction (RERI) and attributable proportion due to interaction (AP) (29). Trend analysis was performed by the Cochran-Armitage trend test with the “DescTools” package (30). To further investigate the specific genetic variants underlying the detected interactions, lifestyle-by-single SNP interaction analysis was conducted with the generalized linear regression model in PLINK software (26). Age, sex, TDI, genotyped batch, assessment center, and the top 10 PCs were used as covariates. Statistical significance was set to a two-sided *P* value of less than 0.05. All statistical analyses were performed in *R* 3.6.1 and PLINK software (version v1.90 b3.38) (31).

## Results

### Basic Characteristics

As shown in **Table 1**, after strict quality control, a total of 3,537 RA patients (2,565 new cases) were included in this study, so the incidence and prevalence of RA were estimated to be 1.3% and 0.9%. We randomly divided the data set into a training set (20%) and a test set (80%) (**Table 1**). Notably, the training set was only used to select the appropriate PRS threshold and fitted prediction models, and all other analyses were performed in the test set. RA patients were excluded at baseline in the prospective analysis, and the follow-up date was updated to June 31, 2021, with a median follow-up of 12 years. The overflow of our research is presented in **Figure 1B**. In addition, we found that the gender-specific participants differed in all lifestyles (**Table S5**), and the prevalence of RA was significantly higher in women than in men (1.59% in women and 0.91% in men). Therefore, we carefully performed gender stratification analyses in most situations.

**Table 1:** Basic characteristics of the study subjects in the UK Biobank cohort.

Variable	All (n = 281,823)		Training Set (N = 56,364)		Test Set (N = 225,459)	
	Control	Case	Control	Case	Control	Case
	278,286 (98.7%)	3,537 (1.3%)	55671 (98.8%)	693 (1.2%)	222,615 (98.7%)	2,844 (1.3%)
Age (mean (SD))	56.25 (8.03)	59.28 (7.14)	56.27 (8.01)	59.20 (7.02)	56.25 (8.03)	59.30 (7.17)
RA onset						
Baseline	/	972 (27.5%)	/	190 (27.4%)	/	782 (27.5%)
Follow-up	/	2,565 (72.5%)	/	503 (72.6%)	/	2062 (72.5%)
sex (%)						
Female	148,578 (53.4)	2,361 (66.8)	29,616 (53.2)	452 (65.2)	118,962 (53.4)	1,909 (67.1)
Male	129,708 (46.6)	1,176 (33.2)	26,055 (46.8)	241 (34.8)	103,653 (46.6)	935 (32.9)
TDI (mean (SD))	-1.65 (2.89)	-1.24 (3.09)	-1.66 (2.89)	-1.13 (3.10)	-1.65 (2.88)	-1.26 (3.09)
Lifestyles						
Regular physical activity	197,501 (71.0)	2,100 (59.4)	39,658 (71.2)	419 (60.5)	157,843 (70.9)	1,681 (59.1)
No smoking	169,790 (61.0)	1,832 (51.8)	33,912 (60.9)	359 (51.8)	135,878 (61.0)	1,473 (51.8)
No alcohol consumption	16,579 (6.0)	413 (11.7)	3,350 (6.0)	84 (12.1)	13,229 (5.9)	329 (11.6)
Healthy diet	93,684 (33.7)	1,250 (35.3)	18,845 (33.9)	258 (37.2)	74,839 (33.6)	992 (34.9)
Moderate BMI	217,418 (78.1)	2,532 (71.6)	43,473 (78.1)	472 (68.1)	173,945 (78.1)	2,060 (72.4)
PRS (%)						
Low risk	70,456 (25.0)	69,849 (25.1)	13,917 (25.0)	121 (17.5)	55,932 (25.1)	486 (17.1)
Intermediate risk	140,911 (50.0)	139,265 (50.0)	27,815 (50.0)	330 (47.6)	111,450 (50.1)	1,316 (46.3)
High risk	70,456 (25.0)	69,172 (24.9)	13,939 (25.0)	242 (34.9)	55,233 (24.8)	1,042 (36.6)
PRS non-MHC (%)						
Low risk	69,834 (25.1)	460 (17.9)	13,819 (24.8)	128 (18.5)	56,004 (25.2)	505 (17.8)
Intermediate risk	139,323 (50.0)	1,263 (49.2)	27,849 (50.0)	333 (48.1)	111,382 (50.0)	1,347 (47.4)
High risk	69,452 (24.9)	842 (32.8)	14,003 (25.2)	232 (33.5)	55,229 (24.8)	992 (34.9)

### Selection of PRS in the Training Set

**Figure 2:**
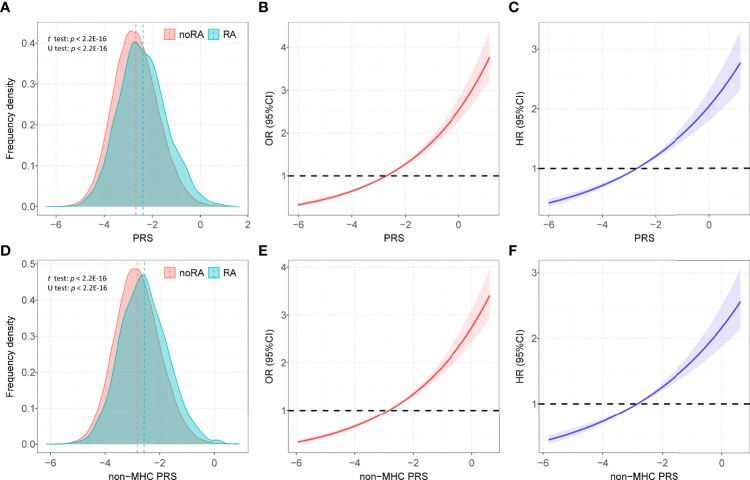
The distributions of PRSs among participants with and without RA. **(A)** Distribution of PRS among participants with and without RA in all participants; **(B)** Restricted cubic spline models for the relationship between PRS and RA risk in case–control analysis; **(C)** Restricted cubic spline models for the relationship between PRS and RA risk in prospective analysis; **(D)** Distribution of PRS _non-MHC_ among participants with and without RA in all participants; **(E)** Restricted cubic spline models for the relationship between non-MHC PRS and RA risk in case-control analysis; **(F)** Restricted cubic spline models for the relationship between non-MHC PRS and RA risk in prospective analysis; Associations in B, C, E, and F were adjusted for age, sex, genotyped batch, assessment center, TDI and the first 10 principal components of ancestry. RA, rheumatoid arthritis; PRS, polygenic risk score; MHC, major histocompatibility complex; OR, odds ratio; HR, hazard ratio.

We calculated 5 PRSs for RA based on SNP information from published GWAS results and genotypes from a large UK Biobank database under different thresholds (*P* < 5×10^-4^, *P* < 5×10^-5^, *P* < 5×10^-6^, *P* < 5×10^-7^, *P* < 5×10^-8^). We used multivariate regression models to assess the associations between these PRSs and RA risk in the training set after adjusting for the covariates. The PRS derived from 80 single-nucleotide polymorphisms (SNPs) (*P* < 5×10^-7^) showed the strongest association with RA risk in the training set (OR = 1.352, 95% CI = 1.257~1.454, *P* = 4.83×10^-16^; HR = 1.216, 95% CI = 1.114~1.328, *P* = 1.22×10^-5^) (**Table S6**), and detailed information of SNPs used was summarized in **Table S7**. The distribution of selected PRSs showed that RA subjects tended to have a higher PRS than those without RA (*P* < 0.001) (**Figures 2A, D** **)**, and similar distributions were observed in the sex-stratified subgroups (**Figure S1** **)**. We performed enrichment analysis for genes mapped by SNP in PRS, and results show these genes were highly related to the mechanism of development and progression of RA (e.g., T cell regulation, lymphocyte activation) (**Figure S2**).

### Association Between PRS and RA Risk in the Test Set

**Figure 3:**
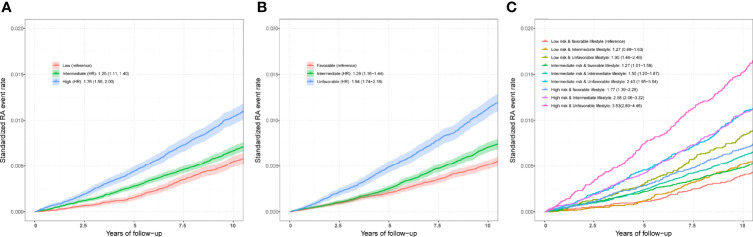
**(A)** Standardized rates of RA events in low (bottom quartile), intermediate (quartiles 2 to 3), and high (top quartile) genetic risk groups in the UKB cohort. **(B)** Standardized rates of RA events in favorable, intermediate, and unfavorable lifestyle groups in the UKB cohort **(C)** Standardized rates of RA events in participates with different genetic risk score groups and different lifestyle groups in the UKB cohort. Associations were adjusted for age, sex, genotyped batch, assessment center, TDI, and the first 10 principal components of ancestry. RA, rheumatoid arthritis; PRS, polygenic risk score; MHC, major histocompatibility complex; OR, odds ratio; HR, hazard ratio. Definition of healthy lifestyle indicators: include 0 to 1 healthy lifestyle indicator for unfavorable lifestyle, 2 for moderate lifestyle, and all 3 for favorable lifestyle. The genetic risk groups were evaluated by PRS: low genetic risk (bottom quartile of PRS), intermediate genetic risk (quartiles 2 to 3), and high genetic risk (top quartile).

After adjusting for covariates, positive associations were observed between best-performed PRS and RA risk in the test set (OR = 1.407, 95% CI = 1.354~1.463, *P* = 7.45×10^-67^; HR = 1.316, 95% CI = 1.257~1.377, *P* = 2.03×10^-32^). Sex-stratified analysis showed that a per SD increase in PRS was significantly associated with RA risk in men (OR = 1.397, 95% CI = 1.306~1.494, *P* = 1.87×10^-22^; HR = 1.344, 95% CI = 1.244~1.452, *P* = 6.09×10^-14^) and women (OR = 1.412, 95% CI = 1.347~1.481, *P* = 5.03×10^-46^; HR = 1.300, 95% CI = 1.229~1.375, *P* = 4.23×10^-20^). From the first decile to the tenth decile, we also found an approximate gradient increase in RA risk (**Figures S3A, B**), and similar results were observed in sex-stratified analyses (**Figures S3C-F**). The restricted cubic spline model was used to evaluate the relationship between PRS and RA risk. In both case-control and prospective studies, PRS and PRS _non-MHC_ were also observed to be related to the risk of RA (**Figures 2B, C, E, F**), and similar results were found for men and women (**Figure S4**). We further divided PRS into three groups (low, intermediate, and high genetic risk). Individuals in the intermediate and high genetic risk groups had a higher risk of RA than those in the low genetic risk group. Compared with the low genetic risk group, the ORs of the intermediate and high groups were estimated to be 1.347 (95% CI = 1.213~1.496, *P* = 2.40×10^-08^) and 2.169 (95% CI = 1.946~2.417, *P* = 1.80×10^-44^), respectively (**Table S8**). In the prospective analysis, the HRs of the intermediate and high groups were estimated to be 1.246 (95% CI = 1.108~1.400, *P* = 2.39×10^-04^) and 1.762 (95% CI = 1.557~1.995, *P* = 3.42×10^-19^), respectively, when compared with the low genetic risk group (**Figures 3A, B** **)**. Similar results were also observed in male and female individuals (**Table S8** and **Figure S5**), as were the results of PRS _non-MHC_ (**Table S9**).

In addition, we evaluated the prediction accuracy of the clinical model (including only age, sex, genotyped batch, TDI, smoking status, physical activity, drinking status, and BMI), PRS + clinical model, and non-MHC PRS + clinical model in the test set. Compared with the non-MHC PRS + clinical model and clinical model, the PRS + clinical model had higher AUCs, with increased AUCs of 0.003 (0.4%) and 0.016 (2.4%) (**Figure S6**).

### Association Between RA Risk and Lifestyle in the Test Set

To define a healthy lifestyle for RA, we considered five possible favorable factors in our analyses: no smoking, no alcohol consumption, moderate BMI, healthy diet, and regular exercise. After correcting for the specified covariates, we found a protective effect of three lifestyles on the risk of RA in the case-control study (**Figure S7**). The OR of RA occurring in subjects with a moderate BMI compared to those with an abnormal BMI was 0.704 (95% CI = 0.639~0.776, *P* = 1.31×10^-12^), and the HR was 0.743 (95% CI = 0.675~0.819, *P* = 2.11×10^-9^). Nonsmokers had a lower risk of RA than smokers (OR = 0.707, 95% CI = 0.647 ~0.772, *P* = 1.57×10^-14^; HR = 0.699, 95% CI = 0.640~0.763, *P* = 1.30×10^-15^). Meanwhile, regular exercise reduced the risk of developing OR (OR = 0.750, 95% CI = 0.685 ~0.821, *P* = 4.77×10^-10^; HR = 0.720, 95% CI = 0.658~0.787, *P* = 5.06×10^-13^), as was the case in both men and women. Furthermore, similar results were observed in the prospective study (**Figure S8**). However, in this large cross-sectional study, we found a protective effect of alcohol consumption on RA, which could not be regarded as a healthy lifestyle. In addition, we did not find a significant association between a healthy diet and the risk of RA.

Finally, we included three favorable factors for further analysis and divided participants into three groups (favorable, intermediate, and unfavorable lifestyles). Compared with those with a favorable lifestyle, logistic regression analysis in a case–control study found an increased risk of RA in individuals adopting intermediate (OR = 1.342, 95% CI = 1.221~1.475, *P* = 9.72×10^-10^) and unfavorable lifestyles (OR = 2.152, 95% CI = 1.950~2.374, *P* = 8.41×10^-53^). In the sex-stratified analysis, the association was weakened in men and enhanced in women. For example, the ORs of unfavorable vs favorable outcomes were estimated to be 2.257 (95% CI = 1.897~2.684, *P* = 3.85×10^-20^) in men and 2.095 (95% CI = 1.859~2.361, *P* = 7.37×10^-34^) in women (**Table S10**). Additionally, in the prospective study, the HRs of unfavorable vs favorable outcomes were estimated to be 1.944 (95% CI = 1.735~2.179, *P* = 3.06×10^-30^) in all participants, 2.030 (95% CI = 1.671~2.467, *P* = 1.02×10^-12^) in men and 1.888 (95% CI = 1.639~2.174, *P* = 1.10×10^-18^) in women (**Table S10**).

### Joint Effect and Interaction of PRS/Genotypes and Lifestyle Factors in the Test Set

**Figure 4:**
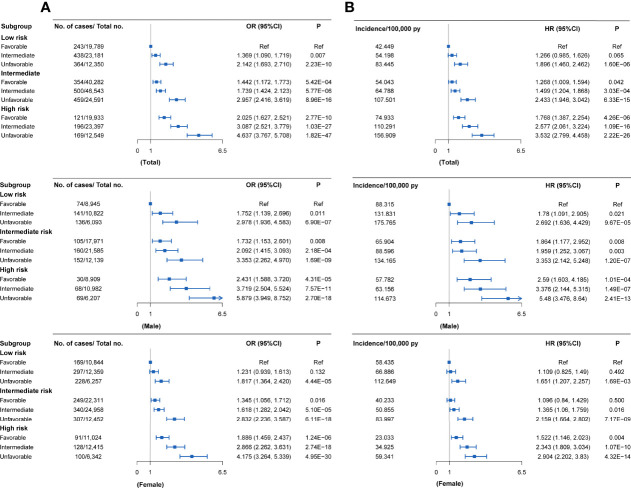
RA risks in the subgroups stratified by genetic PRSs and lifestyles in the UKB cohort (versus participants with favorable lifestyles in the low genetic risk group) **(A)** Case-control study; **(B)** Cohort study. Definition of healthy lifestyle indicators: include 0 to 1 healthy lifestyle indicator for unfavorable lifestyle, 2 for moderate lifestyle, and all 3 for favorable lifestyle. The genetic risk groups were evaluated by PRS: low genetic risk (bottom quartile of PRS), intermediate genetic risk (quartiles 2 to 3), and high genetic risk (top quartile).

Compared with individuals with a low genetic risk and a favorable lifestyle, more than quintuple risks were observed in those with high genetic risk and an unfavorable lifestyle (OR = 4.637, 95% CI = 3.767~5.708, *P* = 1.82×10^-47^; HR = 3.532, 95% CI = 2.799~4.458, *P* = 2.22×10^-26^) (**Figures 3C**,**4A**). A higher association in men (OR = 5.879, 95% CI = 3.949~8.752, *P* = 2.70×10^-18^; HR = 5.480, 95% CI = 3.476~8.640, *P* = 2.41×10^-13^) and a lower risk in women (OR = 4.175, 95% CI = 3.264~5.339, *P* = 4.95×10^-30^; HR = 2.904, 95% CI = 2.202~3.830, *P* = 4.32×10^-14^) were also detected (**Tables S11** and **S12**, **Figure 4A**). In a prospective study, as the genetic risk rises, the risk of RA in patients who adopt a relatively worse lifestyle also increases significantly **(** **Figure 4B** and **Table S11** **)**. Furthermore, similar results were observed in non-MHC PRS analyses (**Tables S13** and **S14**). In all subgroups with different genetic risks or different genders, trend analyses found that the risk of developing RA was associated with increased genetic risk. After considering all five of the widely used lifestyles, although still significant, the association was weakened (**Tables S15** and **S16**). We also evaluated the association in individuals without excluding diseases (**Tables S17** and **S18**). Sensitive analyses were performed after re-selection of training (40%) and test sets (60%) (**Tables S19** and **S20**), after redefining the appropriate BMI as 18.5~25 (**Tables S21** and **S22**), and after redefining the healthy smoking behavior with no current smoking (**Tables S23** and **S24**). All results demonstrate the robustness of the association. In addition, we observed an additive interaction between high genetic risk and healthy lifestyles in both the whole population and sex-stratified groups (**Tables S25** and **S26**). Further lifestyle-by-single SNP interaction analyses found interactions between the 10 RA-associated SNPs and healthy lifestyles (P<0.05), but the significance of the association was relatively low (**Table S27**).

## Discussion

This study constructed the PRS for RA to assess the joint effect of lifestyle and genetics on RA risk in the UKB cohort. The prediction of RA risk was improved by integrating lifestyle and genetic factors. Specifically, high genetic risk, as defined by the PRS, is associated with an increased risk of developing RA in large case-control and prospective studies. Three favorable lifestyles were significantly associated with a reduced risk of RA. Subjects with high genetic risk and unfavorable lifestyles have an increased risk of developing RA compared to those with low genetic risk and favorable lifestyles. Furthermore, adherence to a healthy behavioral lifestyle was associated with a reduced risk of developing RA within each of the different genetic risk groups. To the best of our knowledge, our study is the first to assess the joint effect of lifestyles and genetic factors on RA risk in a large sample.

Three healthy lifestyles were identified to be related to decreased RA risk: tobacco smoking could increase RA risk, which was also proven in previous observational studies (32). A meta-analysis showed that smoking is a risk factor for RA, especially for heavy smokers with RA (33). A population-based case-control study in Sweden showed that gene-environment interactions between smoking and genes increased the risk of RA (34). The involved mechanisms are complex, probably due on the one hand to the effects of smoking on the immune system (e.g., oxidative stress, apoptosis, proinflammatory, and epigenetic effects) and on the other hand to the direct effects of smoking on synovial fibroblasts (10). In addition, the results of the case-control and cohort studies were not identical but showed consistent trends, which increases the reliability of the conclusions.

Some previous studies have also reported the protective effect of moderate alcohol consumption on RA risk. In two large prospective cohorts, moderate alcohol usage had a reduced risk of RA compared to no alcohol consumption, with an HR of 0.78 (95% CI, 0.61-1.00) (35). The mechanism underlying such an association has not been well explained. On the one hand, this may be because alcohol intake increases serum estradiol concentrations, which play an important role in the body’s immune response (36, 37); on the other hand, alcohol has been shown to significantly inhibit the synthesis of proinflammatory cytokines and chemokines (38, 39). A recent animal experiment clarified the immune regulation and tolerance induction effects of drinking (40). Therefore, moderate alcohol consumption may stop or delay the progression of RA. However, given the increased risk of other serious health problems associated with moderate alcohol consumption, we did not regard alcohol consumption as a healthy lifestyle in our study.

In previous studies, even though the association between diet and RA risk was not as strong as other risk factors (e.g., smoking, BMI), its impact on RA has been widely studied. A comprehensive literature review shows the controversy between different dietary approaches and the risk of RA (6). For example, a study has shown that an increased risk of RA is associated with excessive consumption of red meat and high total protein (41). However, a multicenter case-control study showed no significant difference in red meat intake between the two groups (42). In addition, high carbohydrate intake may be associated with an increased risk of RA, while mushrooms, citrus fruits, and dairy products reduce the risk (42). A prospective study based on the Nurses’ Health Study (NHS) shows no association between protein, iron, and meat intake and the risk of RA (43). High dietary sodium (salt) intake is associated with an increased risk of RA and may interact with smoking (44). The high complexity of nutrient supply is also a major factor affecting the composition of the microbiota, which is ultimately involved in the occurrence and development of RA. However, our study did not find a reduction in the risk of RA with a healthy diet, which may be due to healthy volunteer bias in the retrospective investigation. Therefore, more long-term cohort studies are needed to explore this.

In contrast, BMI can be a good indicator of long-term lifestyle as opposed to a record of a healthy diet. Our results found that moderate BMI could lower RA risk, especially in women. A meta-analysis of 13 cohort studies also showed that higher BMI was associated with an increased risk of RA, and this association was limited to women (45). A few potential mechanisms could explain obesity causing RA. First, the obese state is characterized by a low degree of systemic inflammation, and adipose tissue can produce many bioactive molecules that regulate carbohydrate and lipid metabolism, immune function, etc. (46). Second, the prevalence of vitamin D deficiency in obese individuals is high, and such deficiency is also very common in individuals with autoimmune diseases (including RA) (47).

Some SNPs have been shown to interact with lifestyles at risk of RA, and most of the genes mapped to them have been proven to be related to alterable lifestyles in previous studies. For example, compared with sham-exposed controls, mice exposed to cigarette smoke had higher expression of the EOMES gene, indicating the mechanism by which smoking regulates this gene (48). At the same time, EOMES-positive CD4 + T cells proved to be a risk T cell subset of RA (49); Studies have also discussed the molecular role of RUNX1 in obesity and related metabolic diseases (50). However, the statistical significance for each SNP was far from GWAS significance in our result. This is most likely due to the weakening of the association between certain SNPs and RA in the UKB, which is also the limitation of other GWAS studies (less reproducibility) (51). In contrast, our results show that PRS, which represents polygenic effects, can well explain the genetic risk of RA in the UKB cohort. Therefore, we recommend that PRS should be used more clinically for fine division and precise treatment of the population. Previous studies have reported that regular exercise could help to prevent bone loss, strengthen the heart and lungs, and reduce joint pain in RA patients (52, 53). Our results revealed a protective effect of physical exercise on RA risk, and several reasons could explain this association. It is well known that subjects who exercise regularly maintain a healthy body shape. Exercise has no positive effects on the development of RA, and studies have shown that exercise can help reduce the symptoms of tired RA (54, 55).

Our study demonstrates that adherence to a healthy lifestyle reduces the risk of RA within different subgroups, while participants at high genetic risk also benefit from adopting a healthy lifestyle. Interaction analysis also demonstrates the importance of adopting a healthy lifestyle with high genetic risk. The estimated values of additive interaction were greater than zero between high genetic risk and lifestyles, suggesting a positive additive interaction. The risk of simultaneous exposure was higher than the sum of exposure to unhealthy lifestyles or higher genetic risk only. Results of the sex-stratified analysis showed consistent trends, demonstrating that healthy lifestyles are suitable for everyone. In addition, the associations in women were weaker than in men, indicating that the beneficial effects of healthy lifestyles were greater for men than for women. The crucial role of genetic-environmental interactions in the pathogenesis of RA has been well documented in previous studies (17, 34, 56).

When constructing the PRS for RA, different PRSs (*P* range from 5×10^-4^ to 5×10^-7^) may have similar predictive powers, suggesting that except for the SNPs used in our selected PRS, remaining SNPs were not important for the prediction of RA risk. Meanwhile, utilization of PRS with fewer SNPs likewise reduces the cost of practical application in the clinic. In addition, in our analysis, PRSs containing MHC genotypes were more strongly associated with RA and had better predictive power in the validation set. Numerous studies have shown that the main genetic risk factor for RA is the HLA DRB1 allele, while many other non-MHC genes are also associated with RA (3, 57, 58). Enrichment analysis also indicated that the SNPs used for PRS construction matched to genes involved in the progression of RA. Both our results and previous evidence proved the crucial role of MHC genotypes in the development of RA.

Our study observed relatively smaller interaction effect sizes for genetic and environmental factors than previous studies. This inconsistency could be attributed to several factors. First, RA is a complex autoimmune disease determined by multiple genetic variants with relatively small but cumulative effects. Therefore, according to the complex determination nature of the RA, the low interaction effects between SNPs and lifestyles are commonly observed. Second, the accurate definition of lifestyles would be important confounding factors in masking the detection of interaction effects, which would be missed in a small sample size, but be present in the current large sample size. Third, the information on lifestyle was collected at baseline, but the participants’ lifestyles could modify at any time during the long-term follow-up, which might confuse the evaluation of the real interaction effect of lifestyle. Forth, the current study has not fully investigated the underlying causal variation within each gene. For example, rare variants of low frequency may have a significant effect size (increase disease risk by a factor of 2 to 3). These variants are typically missing in both microarrays and 1000 Genomes (containing common variants) (59).

Our study has some limitations. First, lifestyle factors were only assessed at baseline, but the lifestyle of each participant was a dynamic process change. Therefore, more extensive cohort studies are needed to further explore the association with RA risk. Second, only lead SNPs were included in the construction of the PRS, which may have resulted in the loss of polygenic effects. Third, our analysis was based on European ancestry, and these results might not extend to other ancestries.

In summary, our results improved the prediction of RA risk by integrating lifestyle and genetic factors. The results would facilitate the implementation of precision prevention strategies. Genetic-based risk assessment can more accurately determine an individual’s risk of RA and facilitate the release of decisions regarding risk management options. Adopting a healthy lifestyle in subjects at high risk can greatly decrease the risk of developing RA, both for men and women.

## Data Availability Statement

The original contributions presented in the study are included in the article/**Supplementary Material**. Further inquiries can be directed to the corresponding authors.

## Ethics Statement

The studies involving human participants were reviewed and approved by North West Multi-centre Research Ethics Committee (MREC). The patients/participants provided their written informed consent to participate in this study.

## Author Contributions

F-YD, S-FL, and X-HY conceived the design of the study; X-HY, R-RC, and LB obtained the data; X-HY, R-RC, PH, and Y-QY cleared up the datasets; X-HY mainly performed the data analyses; F-YD, S-FL, and X-HY drafted and revised the manuscript, and all authors approved the manuscript and provided relevant suggestions. All authors contributed to the article and approved the submitted version.

## Funding

The study was supported by the Natural Science Foundation of China (81872681 82173529, 82173598 and 82103922), the Science and Technology Project of Suzhou (SS202050, SYS2019024), the QingLan Research Project of Jiangsu Province, and a Project of the Priority Academic Program Development of Jiangsu Higher Education Institutions, Postgraduate Research & Practice Innovation Program of Jiangsu Province (KYCX22_3227).

## Conflict of Interest

The authors declare that the research was conducted in the absence of any commercial or financial relationships that could be construed as a potential conflict of interest.

## Publisher’s Note

All claims expressed in this article are solely those of the authors and do not necessarily represent those of their affiliated organizations, or those of the publisher, the editors and the reviewers. Any product that may be evaluated in this article, or claim that may be made by its manufacturer, is not guaranteed or endorsed by the publisher.
