# Supplementary material for: Systematic Evaluation of Rheumatoid Arthritis Risk by Integrating Lifestyle Factors and Genetic Risk Scores
Source: Front Immunol. 2022 Jul 6;13:901223. doi: 10.3389/fimmu.2022.901223 (PMC9299428; doi:10.3389/fimmu.2022.901223)
Supplement: Supplementary file 1 [file DataSheet_1.docx]

Supplementary file

# Supplementary tables and figures


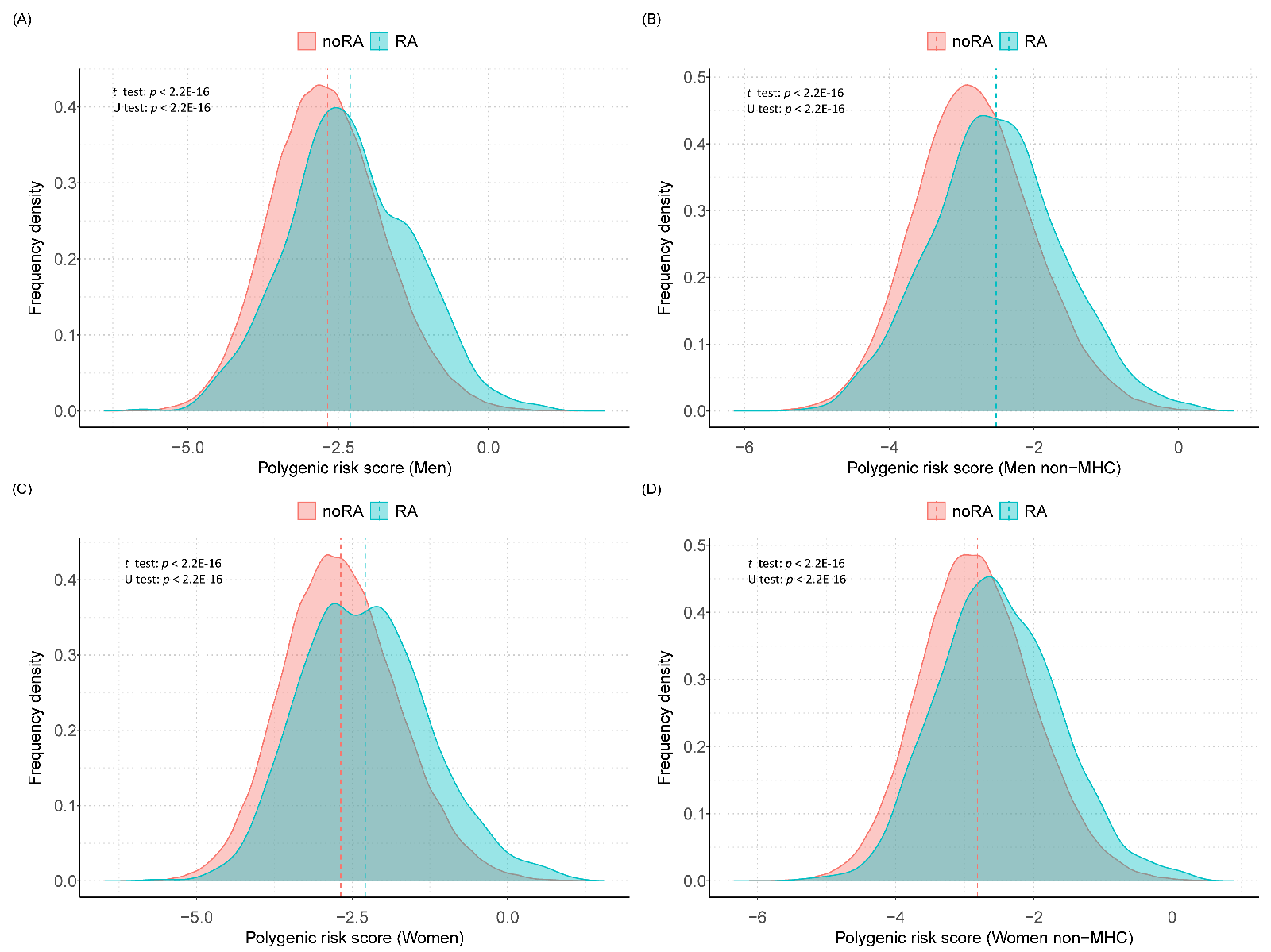


**Figure S1.** The distributions of sex specific PRS between participates with and without RA in all participates. (**A**) Distribution of PRS in men; (**B**) Distribution of PRS _non-MHC_ in men; (**C**) Distribution of PRS in women; (**D**) Distribution of PRS _non-MHC_ in women. The reference lines represent mean of PRS in each group. Abbreviations: RA, rheumatoid arthritis; PRS, polygenic risk score; MHC, major histocompatibility complex.


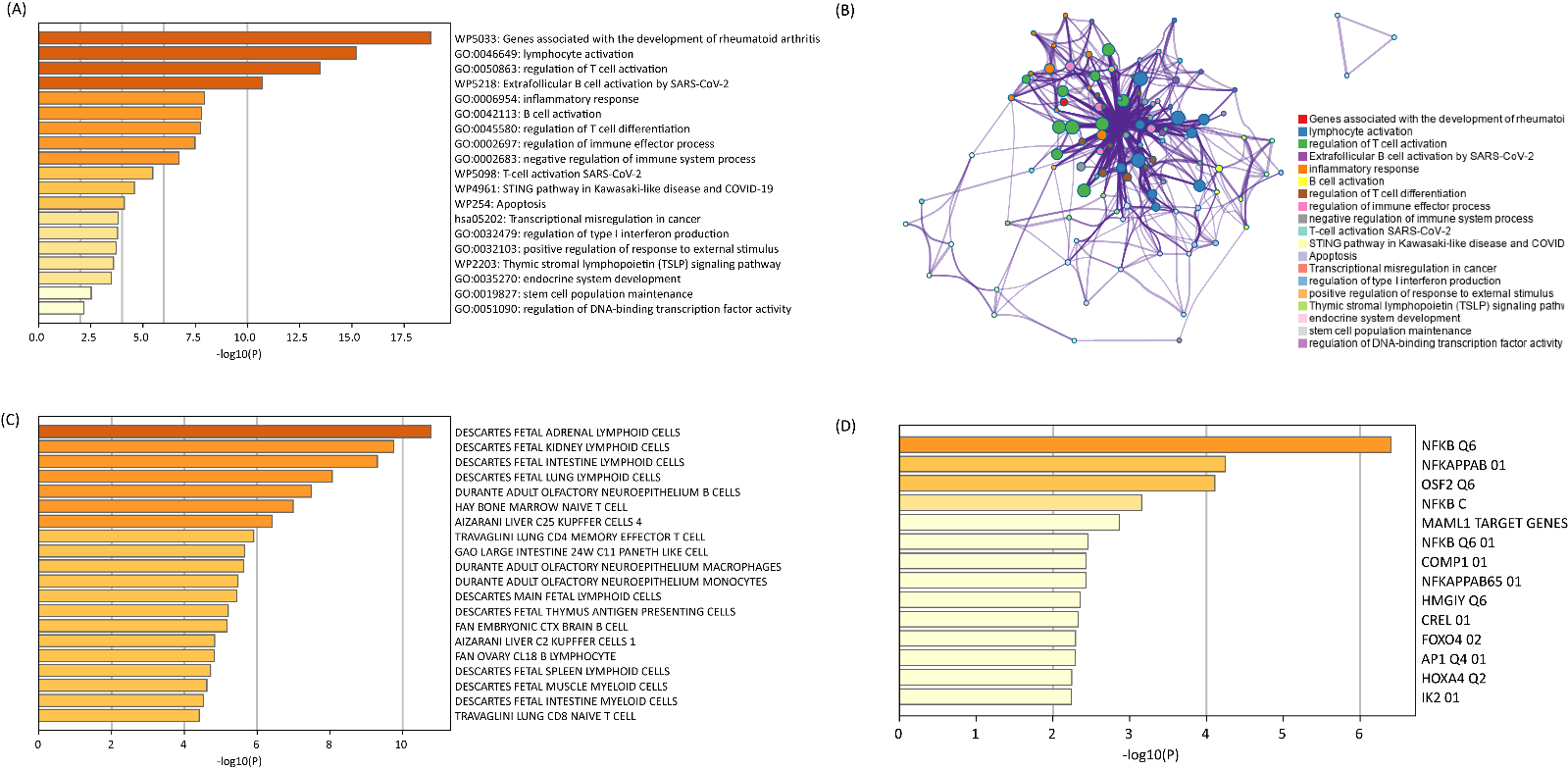


**Figure S2.** Enrichment analysis on mapped genes with SNPs used for construction of PRS (*P* < 5E^-7^). (A) Bar graph of enriched terms across input gene lists, colored by p-values; (B) Network of enriched terms colored by cluster ID, where nodes that share the same cluster ID are typically close to each other; (C) Summary of enrichment analysis in Cell Type Signatures; (D) Summary of enrichment analysis in Transcription Factor Targets.


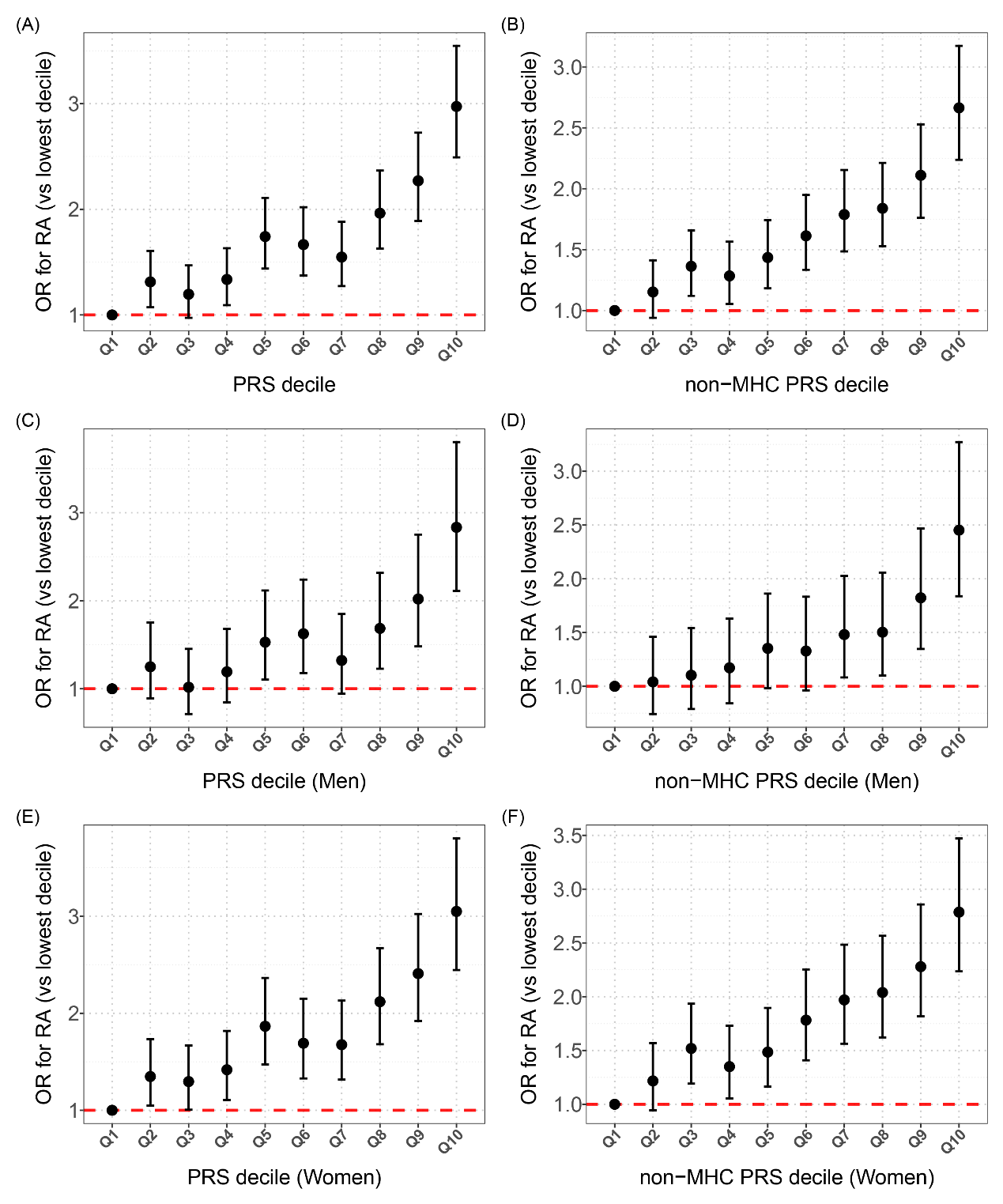


**Figure S3.** Sex-specific decile analysis of PRS on RA risk between the participates with and without RA in test set. (**A**) Decile analysis of PRS on RA risk (versus lowest decile); (**B**) Decile analysis of non-MHC PRS on RA risk (versus lowest decile); (**C**) Decile analysis of PRS on RA risk (versus lowest decile) in men; (**D**) Decile analysis of non-MHC PRS on RA risk (versus lowest decile) in men; (**E**) Decile analysis of PRS on RA risk (versus lowest decile) in women; (**F**) Decile analysis of non-MHC PRS on RA risk (versus lowest decile) in women. Note: Associations were adjusted for age, sex, genotyped batch, assessment center, Townsend deprivation index and the first 10 principal components of ancestry. Abbreviations: RA, rheumatoid arthritis; PRS, polygenic risk score; MHC, major histocompatibility complex; OR, odds ratio.


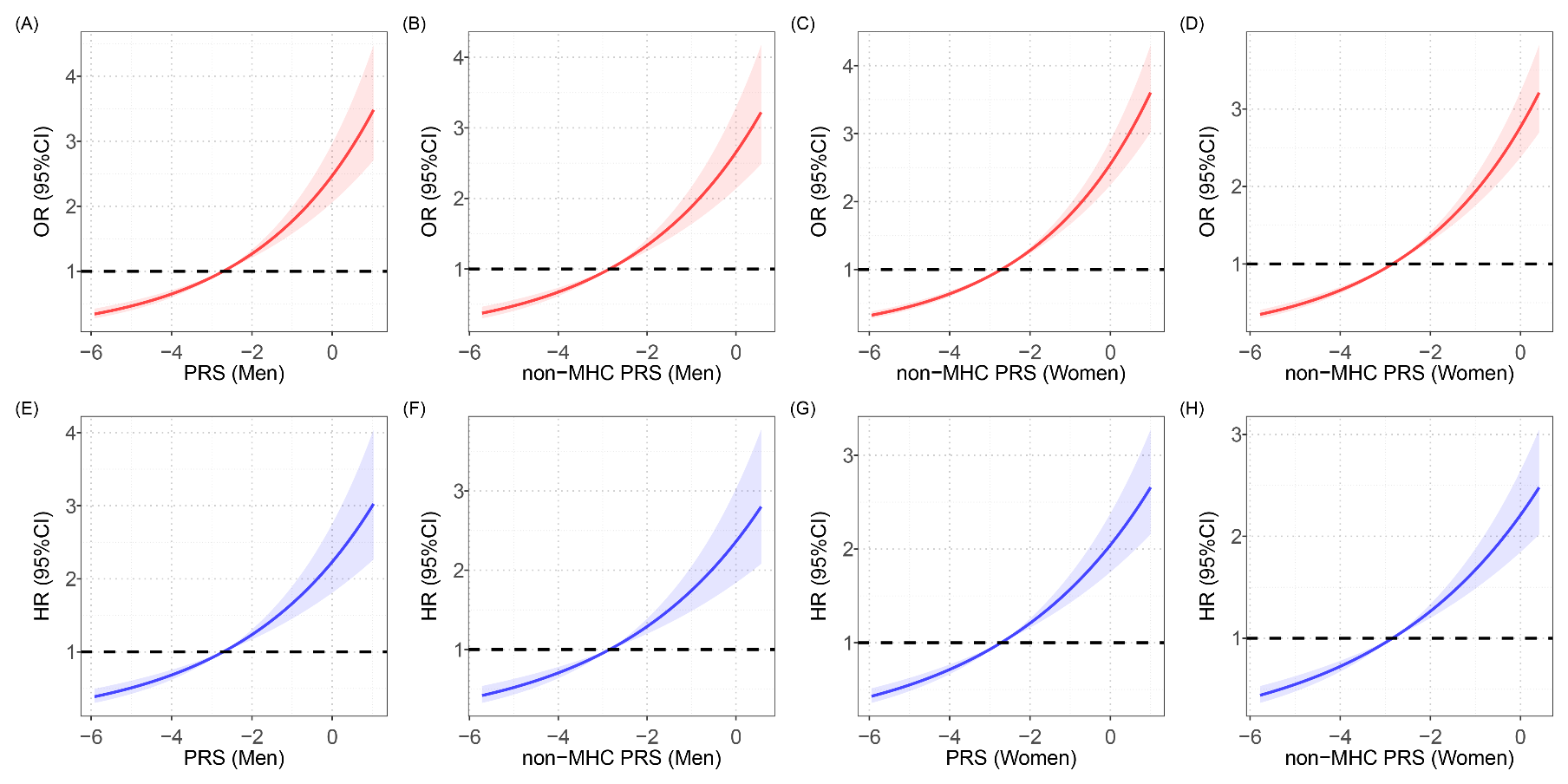


**Figure S4.** (A) Restricted cubic spline models for the relationship between PRS with RA risk for men in case-control analysis; (B) Restricted cubic spline models for the relationship between non-MHC PRS with RA risk for men in case-control analysis; (C) Restricted cubic spline models for the relationship between non-MHC PRS with RA risk for women in case-control analysis; (D) Restricted cubic spline models for the relationship between non-MHC PRS with RA risk for women in case-control analysis; (E) Restricted cubic spline models for the relationship between PRS with RA risk for men in prospective analysis; (F) Restricted cubic spline models for the relationship between non-MHC PRS with RA risk for men in prospective analysis; (G) Restricted cubic spline models for the relationship between PRS with RA risk for women in prospective analysis; (H) Restricted cubic spline models for the relationship between PRS with RA risk for women in prospective analysis; Note: Associations were adjusted for age, sex, genotyped batch, assessment center, Townsend deprivation index and the first 10 principal components of ancestry. Abbreviations: RA, rheumatoid arthritis; PRS, polygenic risk score; MHC, major histocompatibility complex; OR, odds ratio; HR, hazard ratio.


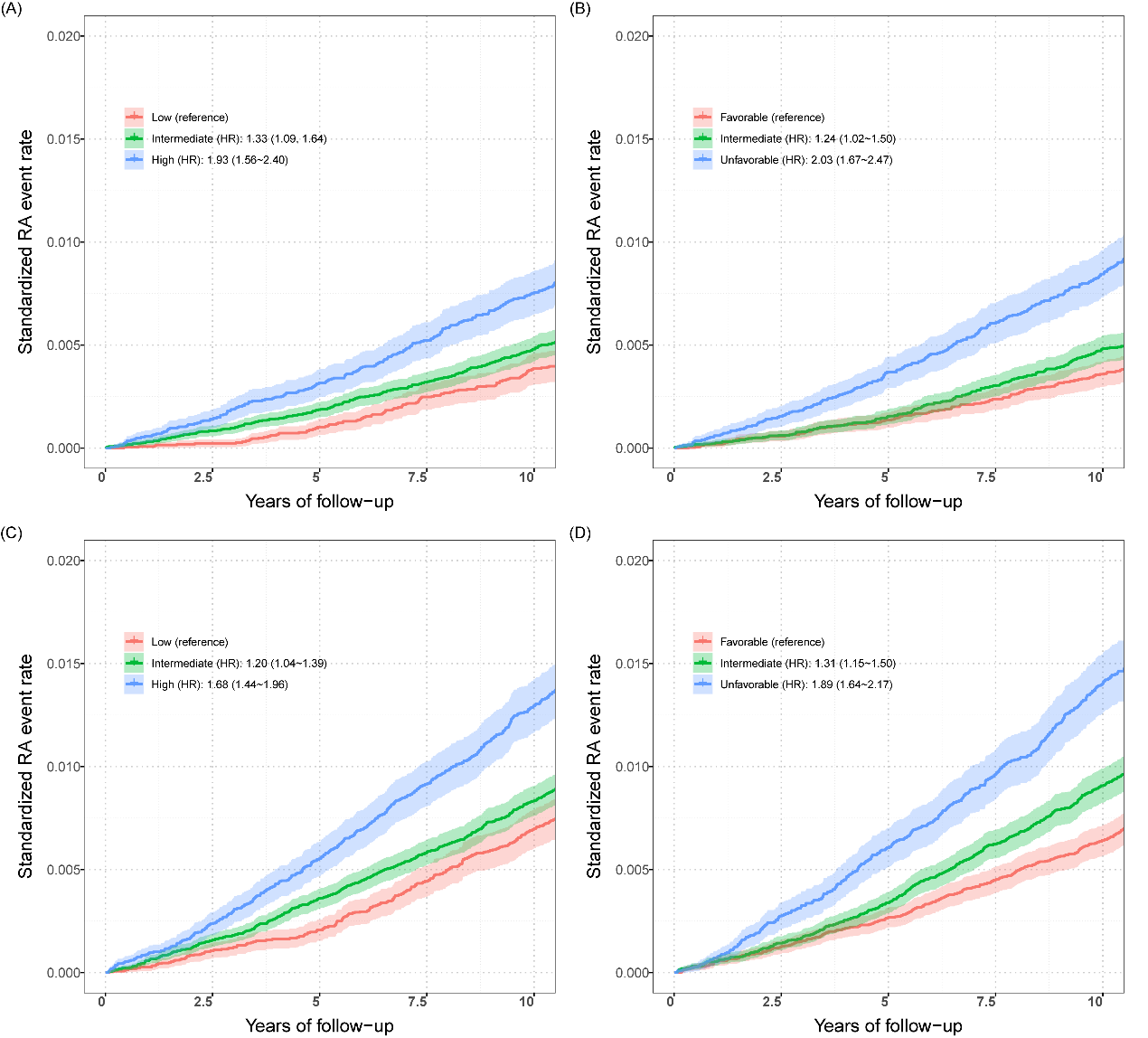


**Figure S5.** (A) Standardized rates of RA events in low (bottom quartile), intermediate (quartiles 2 to 3), and high (top quartile) genetic risk groups in the UKB cohort for men. (B) Standardized rates of RA events in favorable, intermediate, and unfavorable lifestyle groups in the UKB cohort for men; (C) Standardized rates of RA events in low (bottom quartile), intermediate (quartiles 2 to 3), and high (top quartile) genetic risk groups in the UKB cohort for women. (D) Standardized rates of RA events in favorable, intermediate, and unfavorable lifestyle groups in the UKB cohort for women. Note: Associations were adjusted for age, sex, genotyped batch, Townsend deprivation index and the first 10 principal components of ancestry. Note: RA, rheumatoid arthritis; PRS, polygenic risk score; MHC, major histocompatibility complex; OR, odds ratio; HR, hazard ratio. Definition of healthy lifestyle indicators: include 0 to 1 healthy lifestyle indicator for unfavorable lifestyle, 2 for moderate lifestyle, and all 3 for favorable lifestyle. The genetic risk groups were evaluated by PRS: low genetic risk (bottom quartile of PRS), intermediate genetic risk (quartiles 2 to 3) and high genetic risk (top quartile).


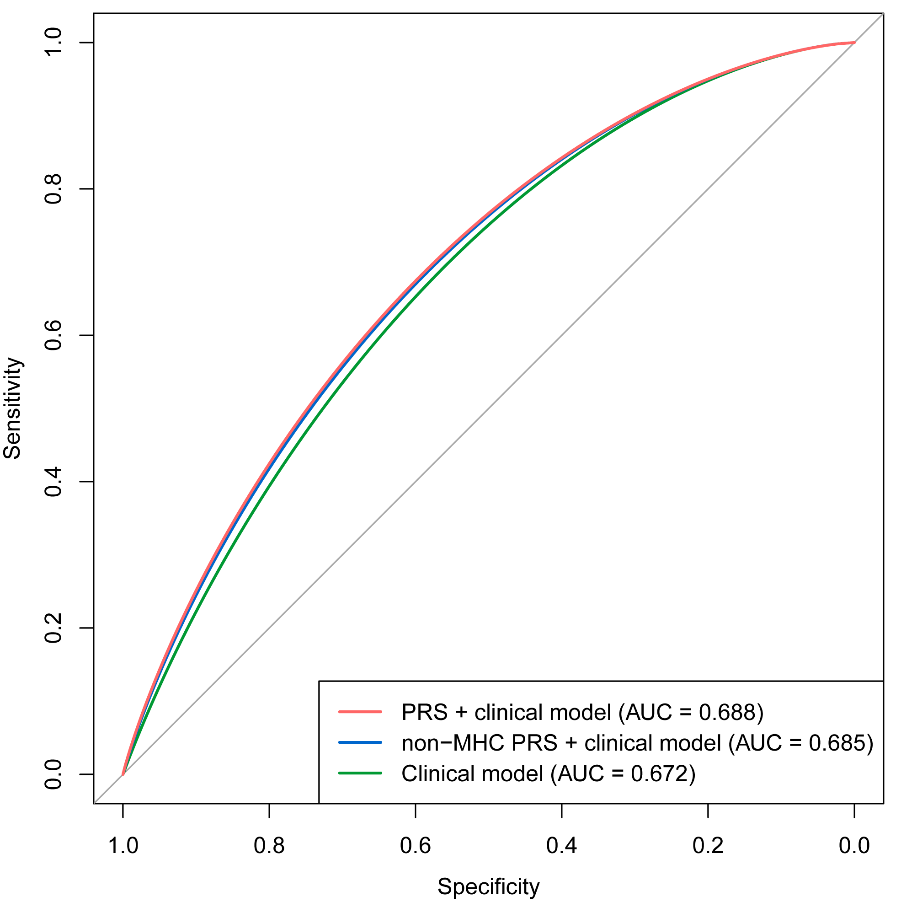


**Figure S6.** Receiver operating characteristic (ROC) curves demonstrating the discriminative performance in test set (i.e., ability to distinguish RA cases from controls) of three PRS models in test data: Clinical model, PRS + clinical model, and PRS _non-MHC_ + clinical model. Note: Clinical model includes age, sex, genotyped batch, Townsend deprivation index, smoking status, physical activity, drinking status and BMI. Abbreviations: RA, rheumatoid arthritis; PRS, polygenic risk score; MHC, major histocompatibility complex; AUC, area under the curve.


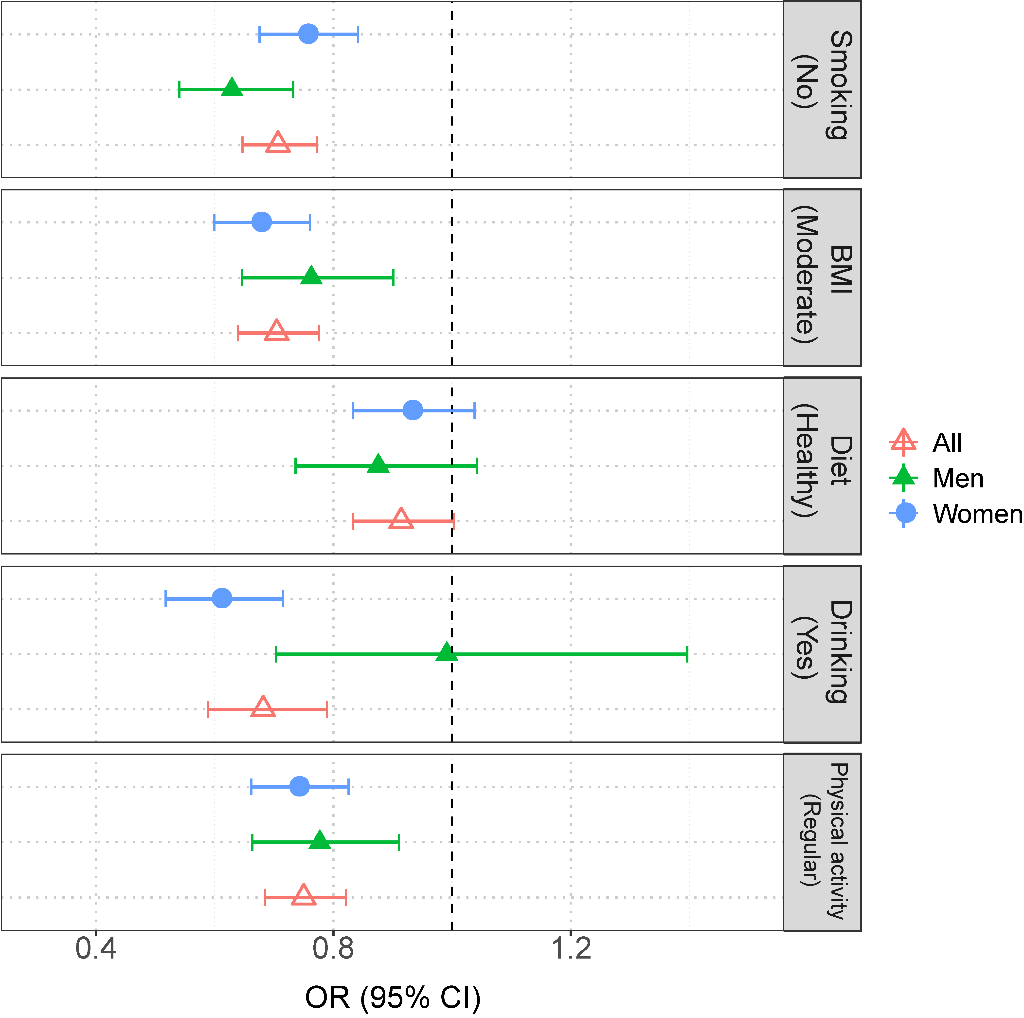


**Figure S7.** Associations between different healthy lifestyles and RA risks in all participates and sex-stratified analysis in case-control study. Smoking, BMI, diet, alcohol drinking and physical activity were shown.


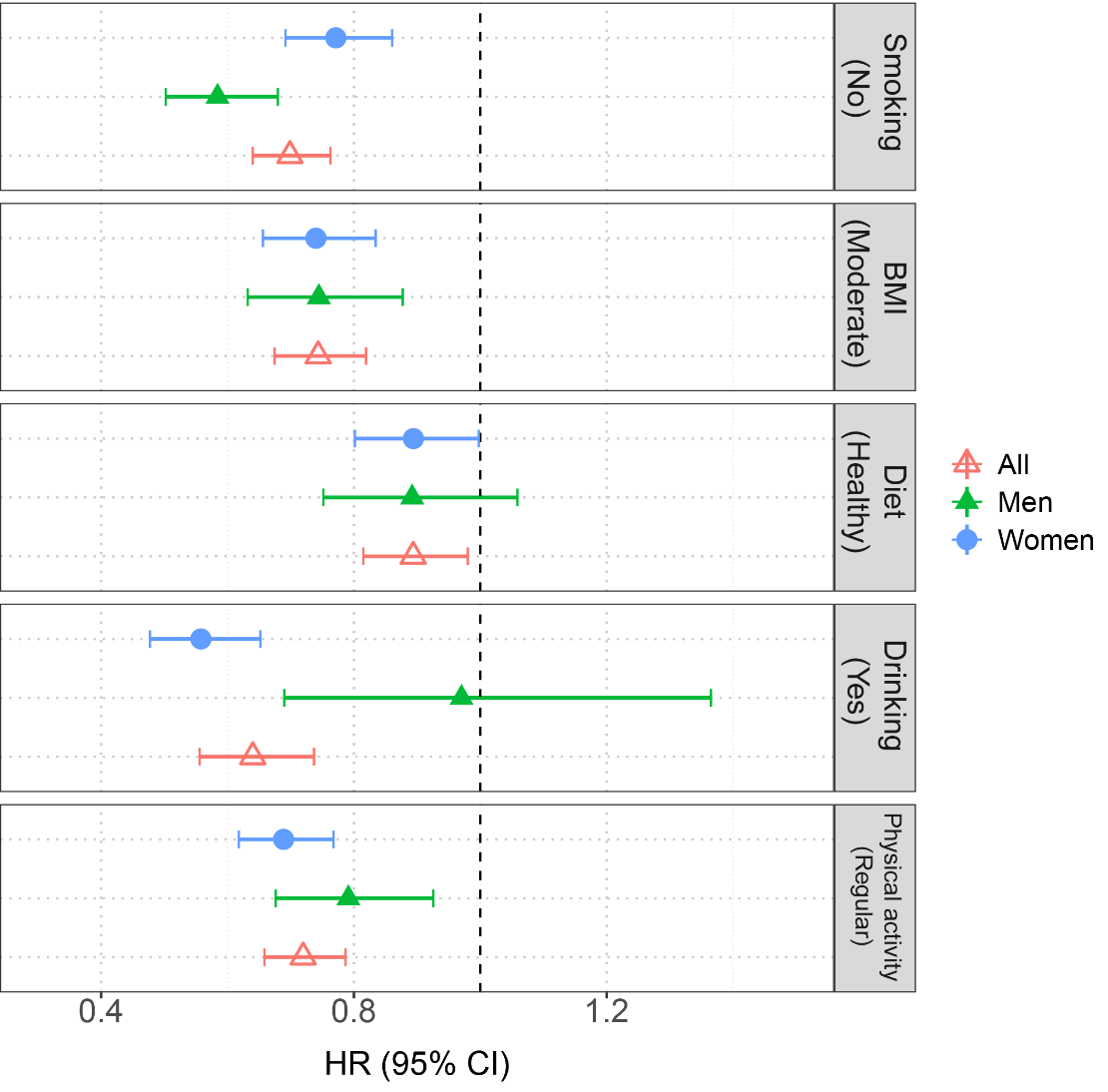


**Figure S8.** Associations between different healthy lifestyles and RA risks in all participates and sex-stratified analysis in prospective study. Smoking, BMI, diet, alcohol drinking and physical activity were shown.

**Table S1. Literature review of the relationship between lifestyle factors and RA in previous studies**

| **Year** | **First author** | **Study design** | **Country** | **Cases/cohort size or controls** | **Highest group** | **HR/RR/OR (95% CI)** | **PMID** |
| --- | --- | --- | --- | --- | --- | --- | --- |
| 1994 | L F Voigt | Case-control | USA | 349/1,457 | Pack-years of smoking: >20 vs ≤20 Alcoholic drinks per week：>14 vs ≤14 BMI: highest quartile vs lowest | 1.49 (1.06 to 2.10) 0.50 (0.20 to 1.70) 1.40 (1.00 to 2.00) | 7986867 |
| 2001 | D Hutchinson | Case-control | UK | 239/239 | Pack-years of smoking: >50 vs ≤50 Ever smoker: Yes vs No | 8.41 (2.45 to 28.84) 1.81 (1.22 to 2.19) | 11171682 |
| 2001 | A Reckner Olsson | Case-control | Sweden | Men 91/203 Women 154/222 | Pack-years of smoking: ≥20 vs Non-smokers | Men 2.7 (1.2 to 5.8) Women 1.8 (0.7 to 4.6) | 11557649 |
| 2002 | Lindsey A Criswell | Cohort | USA | 158/31,336 | Current smoker vs Non-smokers | 2.00 (1.30 to 2.90) | 11959057 |
| 2003 | P Stolt | Case-control | Sweden | 486/724 | Current smoker vs Non-smokers | 1.80 (1.30 to 2.60) | 12922955 |
| 2006 | Karen H Costenbader | Cohort | USA | 680/103,818 | Current smoker, Former smoker vs Non-smokers | 1.42 (1.16 to 1.75) and 1.47 (1.23 to 1.76) | 16750964 |
| 2006 | Merete Pedersen | Case-control | Denmark | 515/769 | Current smoker, Former smoker vs Non-smokers | 1.80 (1.37 to 2.36) and 1.57 (1.13 to 2.19) | 16872514 |
| 2010 | Ted R Mikuls | Case-control | USA | 605/255 | Current smoker, Former smoker vs Non-smokers | 1.56 (1.07 to 2.26) and 1.45 (1.07 to 1.97) | 20722010 |
| 2012 | Abqariyah Yahya | Case-control | Malaysia | 1056/1416 | Pack-years of smoking:≥20 vs ≤20 | 2.30 (1.00 to 5.50) | 22006120 |
| 2013 | Daniela Di Giuseppe | Cohort | Swedish | 219/34,101 | Pack-years of smoking: >22 vs ≤22 | 1.82 (1.19 to 2.79) | 23607815 |
| 2014 | Bing Lu | Cohort | USA | 1181/218623 | Obese vs Normal (women) | 1.37 (0.91 to 2.09) | 25057178 |
| 2015 | Eva Salgado | Case-control | Spain | 392/18555 | Total sodium intake: highest quartile vs Lowest | 1.50 (1.10 to 2.10) | 26376372 |
| 2015 | Daniela Di Giuseppe | Cohort | Swedish | 201/30112 | Category of leisure-time activity: Highest vs Lowest | 0.65 (0.43 to 0.96) | 25884929 |
| 2019 | Xinyi Liu | Cohort | USA | 506/113366 | Recreational physical activity: ≥7 hours/week vs <1 hour/week | 0.67 (0.47 to 0.98) | 30920773 |
| 2020 | Louise Hedenstierna | Cohort | Swedish | 577/41068 | Alcohol consumption: High vs None Smoker vs non-smoker in non-drinkers and alcohol consumers | 0.54 (0.41 to 0.73) 2.80 (1.96 to 4.01) and 1.45 (1.21 to 1.74) | 33414179 |
| 2020 | Shokufeh Nezamoleslami | Case-control | Iran | 100/297 | Healthy dietary pattern: Low vs High Western dietary pattern: High vs Low | 2.85 (1.12 to 7.45) 2.22 (1.04 to 4.72) | 32963579 |

**Table S2. Literature review of previous studies about genetic risk scores for RA**

| **Years** | **First author** | **Title** | **Sample size** | ***n*** | **Main results** | **PMID** |
| --- | --- | --- | --- | --- | --- | --- |
| 2011 | Lori B Chibnik | Genetic risk score predicting risk of rheumatoid arthritis phenotypes and age of symptom onset | 542 RA cases and 551 controls | 39 | Comparing the highest GRS risk group to the intermediate risk group, we found an OR of 1.2 (95% CI = 0.8-2.1) for seronegative RA, 3.0 (95% CI = 1.9-4.7) for seropositive RA, 3.2 (95% CI = 1.8-5.6) for erosive RA, and 7.6 (95% CI = 3.6-16.3) for seropositive, erosive RA | 21931699 |
| **SNP used:** rs10488631, rs10919563, rs11586238, rs11676922, rs13315591, rs1678542, rs17066662, rs1980422, rs2240340, rs2476601, rs26232, rs2812378, rs3087243, rs3093023, rs3761847, rs3890745, rs394581, rs42041, rs4750316, rs4810485, rs540386, rs548234, rs6822844, rs6859219, rs6920220, rs706778, rs7552317, rs7574865, rs874040, rs934734, rs951005, DRB*0401, DRB*0404, DRB*0405, DRB*0408, DRB*0101, DRB*0102, DRB*1001, DRB*09 | | | | | | |
| 2013 | Sampath Prahalad | Susceptibility to childhood-onset rheumatoid arthritis: investigation of a weighted genetic risk score that integrates cumulative effects of variants at five genetic loci | 155 children with childhood-onset RA and 684 healthy controls | 5 | There was a significant difference in GRS between RA cases and controls (P < 2 × 10(-16) ). Individuals in the third to fifth quintile of GRS had a significantly increased risk of disease compared with the reference group (individuals in the first quintile). In addition, higher GRS was associated with an increased risk of RA in childhood, especially in males. | 23450725 |
| **SNP used:** rs2476601, rs7574865, rs10499194, rs6920220, rs3761847 | | | | | | |
| 2015 | Annie Yarwood | A weighted genetic risk score using all known susceptibility variants to estimate rheumatoid arthritis risk | 11 366 RA cases and 15 489 healthy controls | 46 | Individuals in the highest risk group had significantly increased odds of developing anti-cyclic citrullinated peptide positive RA compared to the lowest risk group (OR 27.13, 95% CI 23.70 to 31.05). The GRS demonstrated better predictive power for RA risk (area under the curve 0.78, OR=18.00, 95% CI=13.67 to 23.71). | 24092415 |
| **SNP used:** rs2843401, rs2240336, rs883220, rs2476601, rs798000, rs10494360, rs2014863, rs8192284, rs34695944, rs6546146, rs10209110, rs13426947, rs1980422, rs11571302, rs35677470, rs932036, rs78560100, rs71624119, rs39984, rs6911690, rs6920220, rs629326, rs59466457, rs3807306, rs4840565, rs2812378, rs10739580, rs10795791, rs947474, rs2275806, rs12764378, rs570676, rs595158, rs4938573, rs10683701, rs8043085, rs8026898, rs13330176, rs12936409, rs34536443, rs6032662, rs2834512, rs9979383, rs3218251, rs13397 | | | | | | |
| 2016 | Xia Jiang | A genetic risk score composed of rheumatoid arthritis risk alleles, HLA-DRB1 haplotypes, and response to TNFi therapy - results from a Swedish cohort study | 2785 RA cases and 1941 controls | 76 | Both the linear and categorical GRS only consistently increased ACPA-positive RA risk but not ACPA-negative RA risk | 27912794 |
| **SNP used:** rs2843401, rs2240336, rs2306627, rs883220, rs2476601, rs798000, rs8192284, rs3761959, rs10494360, rs1557121, rs2014863, rs1355208, rs34695944, rs6546146, rs10209110, rs13426947, rs16837131, rs1980422, rs11571302, rs7639882, rs3806624, rs35677470, rs932036, rs2352593, rs78560100, rs71624119, rs39984, rs10065782, rs660895, rs7764323, rs6911690, rs9386514, rs6920220, rs629326, rs34046992, rs67250450, rs4272, rs3807306, rs4840565, rs998731, rs678347, rs6651252, rs2812378, rs10739580, rs10795791, rs947474, rs2275806, rs793108, rs2671692, rs12764378, rs570676, rs595158, rs968567, rs4409785, rs4938573, rs10683701, rs4766578, rs7993214, rs911263, rs8043085, rs8026898, rs17606153, rs13330176, rs1877030, rs12936409, rs34794968, rs34536443, rs6032662, rs2834512, rs9979383, rs1893592, rs2236668, rs11089637, rs3218251, rs909685, rs13397 | | | | | | |
| 2020 | S Rostami | Comparison of methods to construct a genetic risk score for prediction of rheumatoid arthritis in the population-based Nord-Trøndelag Health Study, Norway | 489 RA cases and 61584 controls | 27 | Increased GRS per SD was associated with RA risk [OR 1.86 (95% CI 1.71, 2.04), p < 0.001]. The AUC for prediction of RA was 0.76 (95% CI 0.74, 0.78). Positive and negative predictive values were 1.6% and 99.7%, respectively | 31930319 |
| **SNP used:** rs2843401, rs2240336, rs6679677, rs624988, rs2105325, rs6732565, rs7574865, rs1980422, rs3087243, rs4452313, rs3806624, rs13315591, rs71624119, rs26232, rs67998226, rs6457617, rs72928038, rs2327832, rs10499194, rs7752903, rs2451258, rs2736340, rs1516971, rs773125, rs10774624, rs8026898, rs34536443 | | | | | | |

Note: *n* represents the number of SNPs used in genetic risk scores. RA: rheumatoid arthritis

**Table S3.** Field IDs used in this research.

| **Variables** | **ID** | **Note** |
| --- | --- | --- |
| Genotype measurement batch | 22000 | Genotyping was done using two closely related arrays, referred to as the UK BiLEVE array and the UK Biobank Axiom array. |
| Assessment center | 54 | The UK Biobank assessment center at which participant consented. |
| Date | 53 | Date of attending assessment center |
| Kinship | 22021 | Genetic kinship to other participants |
| Age | 21003 | Age when attended assessment center |
| gender | 31 | Sex of participant. |
| Townsend deprivation index | 189 | Townsend deprivation index at recruitment |
| Race | 21000 | Ethnic background |
| Genetic principal components | 22009 | Score for each principal component 1-40 |
| ICD10 | 41270 | This field is a summary of the distinct diagnosis codes a participant has had recorded across all their hospital inpatient records in either the primary or secondary position. Diagnoses are coded according to the International Classification of Disease version 10 (ICD-10). |
| ICD10 date | 41280 | Date of first in-patient diagnosis |
| Lifestyles | 20116, 2897, 884, 894, 904,  914,1309, 1319, 1289, 1299, 1329, 1339, 1349, 1369, 1379, 1389, 1438, 1448, 1458, 1468, 20117, 1558, 21001 | See **Table S2**. |

**Table S4.** Definition of healthy lifestyle factor

| **Healthy lifestyle factor** | **Source and definition** | **UK Biobank field codes** |
| --- | --- | --- |
| No current smoking | Former smokers (smoking status) and who have quit smoking for more than 30 years are defined as no current smokers. | 20116, 2897 |
| Regular physical activity | Regular physical exercise is defined as ≥ 150 minutes of moderate intensity activity per week, ≥ 75 minutes of vigorous exercise per week, at least 5 days of moderate intensity exercise per week and one vigorous exercise session per week | 884, 894, 904, 914 |
| Healthy diet | A healthy diet is defined as having four of the six food groups, eating ≥4 servings of fruit, ≥4 servings of vegetables, ≥3 servings of whole grains, ≥2 servings of fish, ≤1 serving of processed meat and ≤1.5 servings of unprocessed red meat per week | 1309, 1319, 1289, 1299, 1329, 1339, 1349, 1369, 1379, 1389, 1438, 1448, 1458, 1468 |
| Alcohol consumption | "Alcohol consumption" was defined as those who drank more frequently than once per month and those whose drinking status was current drinking. | 20117, 1558 |
| BMI moderate | 18.5 ≤ BMI ≤ 30 | 21001 |

**Table S5.** Basic information (gender-stratified) in test set.

|  | | Women | | Men | |
| --- | --- | --- | --- | --- | --- |
|  |  | N | % | N | % |
| **Lifestyles** | |  |  |  |  |
| Regular physical activity | | 83390 | 69.00% | 76134 | 72.80% |
| No smoking | | 77676 | 64.30% | 59675 | 57.10% |
| No drinking | | 8743 | 7.20% | 4815 | 4.60% |
| Healthy diet | | 48711 | 40.30% | 27120 | 25.90% |
| Moderate BMI | | 95686 | 79.20% | 80319 | 76.80% |
| **Lifestyle classification** | |  |  |  |  |
| Favorable lifestyle | | 44688 | 37.00% | 36034 | 34.50% |
| Intermediate lifestyle | | 50497 | 41.80% | 43758 | 41.80% |
| Unfavorable lifestyle | | 25686 | 21.30% | 24796 | 23.70% |
| **PRS classification** | |  |  |  |  |
| High PRS | | 30154 | 24.90% | 26211 | 25.10% |
| Intermediate PRS | | 60617 | 50.20% | 52112 | 49.80% |
| Low PRS | | 30100 | 24.90% | 26265 | 25.10% |
| **Lifestyle* PRS classification** | |  |  |  |  |
| Favorable lifestyle | High PRS | 11013 | 9.10% | 9019 | 8.60% |
| Intermediate lifestyle | High PRS | 12656 | 10.50% | 10963 | 10.50% |
| Unfavorable lifestyle | High PRS | 6485 | 5.40% | 6229 | 6.00% |
| Favorable lifestyle | Intermediate PRS | 22560 | 18.70% | 18076 | 17.30% |
| Intermediate lifestyle | Intermediate PRS | 25298 | 20.90% | 21745 | 20.80% |
| Unfavorable lifestyle | Intermediate PRS | 12759 | 10.60% | 12291 | 11.80% |
| Favorable lifestyle | Low PRS | 11115 | 9.20% | 8939 | 8.50% |
| Intermediate lifestyle | Low PRS | 12543 | 10.40% | 11050 | 10.60% |
| Unfavorable lifestyle | Low PRS | 6442 | 5.30% | 6276 | 6.00% |

**Table S6.** Associations between different PRSs and RA risks

| **Cut-off** | | **N*_SNP_*** | **Training set** | | **Test set** | | **Training set** | | **Test set** | |
| --- | --- | --- | --- | --- | --- | --- | --- | --- | --- | --- |
|  |  |  | **OR (95%CI)** | ***P*** | **OR (95%CI)** | ***P*** | **HR (95%CI)** | ***P*** | **HR (95%CI)** | ***P*** |
| With-MHC PRS | | | | | | |  |  |  |  |
|  | 5.0E-04 | 673 | 1.283 (1.191, 1.383) | 5.79E-11 | 1.174 (1.148, 1.202) | 5.01E-43 | 1.103 (1.009, 1.205) | 0.031 | 1.160 (1.129, 1.191) | 1.02E-27 |
|  | 5.0E-05 | 207 | 1.300 (1.207, 1.400) | 4.03E-12 | 1.312 (1.270, 1.355) | 2.44E-61 | 1.216 (1.113, 1.328) | 1.38E-05 | 1.255 (1.209, 1.302) | 1.06E-32 |
|  | 5.0E-06 | 116 | 1.336 (1.242, 1.438) | 9.77E-15 | 1.385 (1.335, 1.437) | 7.04E-68 | 1.212 (1.111, 1.323) | 1.55E-05 | 1.342 (1.283, 1.404) | 1.54E-37 |
|  | **5.0E-07** | **80** | **1.352 (1.257, 1.454)** | **4.83E-16** | **1.407 (1.354, 1.463)** | **7.45E-67** | **1.216 (1.114, 1.328)** | **1.22E-05** | **1.329 (1.274, 1.387)** | **3.52E-39** |
|  | 5.0E-08 | 57 | 1.149 (1.069, 1.234) | 1.45E-04 | 1.489 (1.347, 1.646) | 6.87E-15 | 1.057 (0.970, 1.153) | 0.208 | 1.380 (1.227, 1.552) | 7.47E-08 |
| Non-MHC PRS | | | | | | |  |  |  |  |
|  | 5.0E-04 | 658 | 1.282 (1.19, 1.382) | 6.25E-11 | 1.175 (1.148, 1.203) | 7.28E-43 | 1.096 (1.003, 1.197) | 0.043 | 1.160 (1.129, 1.191) | 2.04E-27 |
|  | 5.0E-05 | 194 | 1.292 (1.200, 1.391) | 1.25E-11 | 1.311 (1.269, 1.355) | 1.41E-59 | 1.203 (1.101, 1.314) | 3.98E-05 | 1.253 (1.207, 1.302) | 1.73E-31 |
|  | 5.0E-06 | 104 | 1.302 (1.210, 1.401) | 1.99E-12 | 1.380 (1.328, 1.435) | 2.24E-59 | 1.205 (1.104, 1.316) | 2.94E-05 | 1.347 (1.280, 1.418) | 1.94E-30 |
|  | 5.0E-07 | 68 | 1.296 (1.205, 1.395) | 4.26E-12 | 1.424 (1.363, 1.488) | 2.19E-56 | 1.219 (1.117, 1.331) | 8.61E-06 | 1.319 (1.261, 1.380) | 2.19E-33 |
|  | 5.0E-08 | 46 | 1.076 (0.999, 1.159) | 0.053 | 1.409 (1.124, 1.766) | 0.003 | 1.011 (0.925, 1.104) | 0.811 | 1.271 (0.977, 1.654) | 0.074 |

Note: Associations were adjusted for age, sex, genotyped batch, assessment center, Townsend deprivation index and the first 10 principal components of ancestry. Abbreviations: CI, confidence interval; OR, odds ratio; PRS, polygenic risk score; MHC, major histocompatibility complex.

**Table S7. Details on the SNPs used to construct the PRS (*P* < 5E-7).**

| **SNP** | **Chromosome** | **Position** | **Effect Allele** | **Beta** | **Se** | ***P*** |
| --- | --- | --- | --- | --- | --- | --- |
| rs9296009 | 6 | 32,114,515 | A | -0.598 | 0.019 | 1.00E-250 |
| rs2858329 | 6 | 32,658,801 | A | -0.478 | 0.017 | 4.90E-204 |
| rs2476601 | 1 | 114,377,568 | A | 0.593 | 0.023 | 1.60E-149 |
| rs35590025 | 6 | 30,992,111 | T | 0.554 | 0.027 | 9.50E-102 |
| rs9277398 | 6 | 33,051,280 | T | 0.351 | 0.018 | 1.30E-86 |
| rs9276921 | 6 | 32,881,502 | A | 0.501 | 0.029 | 4.30E-67 |
| rs35266973 | 6 | 31,565,384 | A | -0.329 | 0.022 | 1.50E-66 |
| rs4713242 | 6 | 29,718,220 | A | 0.231 | 0.016 | 2.00E-44 |
| rs622917 | 6 | 33,702,869 | T | -0.163 | 0.018 | 8.40E-26 |
| rs7731626 | 5 | 55,444,683 | A | -0.198 | 0.019 | 7.90E-23 |
| rs114561028 | 6 | 31,037,296 | A | -0.528 | 0.055 | 8.50E-22 |
| rs3087243 | 2 | 204,738,919 | A | -0.139 | 0.018 | 9.20E-20 |
| rs17264332 | 6 | 138,005,515 | A | -0.163 | 0.018 | 7.10E-19 |
| rs11751928 | 6 | 28,335,378 | T | -0.198 | 0.026 | 7.60E-19 |
| rs8026898 | 15 | 69,991,417 | A | 0.148 | 0.018 | 2.40E-17 |
| rs13204986 | 6 | 31,100,275 | G | 0.239 | 0.029 | 3.20E-17 |
| rs74956615 | 19 | 10,427,721 | A | -0.371 | 0.046 | 3.30E-16 |
| rs10790268 | 11 | 118,729,391 | A | -0.163 | 0.018 | 3.30E-15 |
| rs72507814 | 6 | 29,541,566 | A | 0.315 | 0.039 | 3.60E-15 |
| rs1571878 | 6 | 167,540,842 | T | -0.117 | 0.017 | 4.90E-15 |
| rs4239702 | 20 | 44,749,251 | T | -0.139 | 0.012 | 4.20E-14 |
| rs86715 | 6 | 33,480,435 | A | 0.182 | 0.022 | 1.10E-13 |
| rs10972201 | 9 | 34,707,373 | A | 0.122 | 0.018 | 1.80E-13 |
| rs12764378 | 10 | 63,800,004 | A | 0.131 | 0.018 | 1.90E-13 |
| rs75218946 | 6 | 26,131,810 | A | 0.385 | 0.051 | 3.80E-13 |
| rs13426947 | 2 | 191,933,254 | A | 0.131 | 0.018 | 2.40E-12 |
| rs8032939 | 15 | 38,834,033 | T | -0.117 | 0.017 | 2.40E-12 |
| rs9653442 | 2 | 100,825,367 | T | -0.105 | 0.017 | 3.60E-12 |
| rs3778753 | 7 | 128,580,042 | A | -0.117 | 0.012 | 4.20E-12 |
| rs706778 | 10 | 6,098,949 | T | 0.104 | 0.014 | 7.10E-12 |
| rs13120727 | 4 | 26,111,351 | A | -0.128 | 0.018 | 9.20E-12 |
| rs185282699 | 6 | 31,505,578 | T | 0.405 | 0.061 | 3.10E-11 |
| rs9603608 | 13 | 40,318,819 | A | 0.104 | 0.014 | 7.60E-11 |
| rs537544 | 10 | 8,108,382 | T | -0.117 | 0.017 | 8.00E-11 |
| rs187786174 | 1 | 2,523,811 | A | -0.117 | 0.017 | 2.20E-10 |
| rs4452313 | 3 | 17,047,032 | A | -0.105 | 0.017 | 2.70E-10 |
| rs2561477 | 5 | 102,608,924 | A | -0.105 | 0.017 | 5.20E-10 |
| rs2451258 | 6 | 159,506,600 | T | 0.104 | 0.019 | 6.60E-10 |
| rs55945621 | 2 | 65,615,354 | A | -0.105 | 0.017 | 1.10E-09 |
| rs2240336 | 1 | 17,674,402 | T | -0.105 | 0.017 | 1.40E-09 |
| rs137687 | 22 | 39,740,078 | A | -0.094 | 0.017 | 1.40E-09 |
| rs59716545 | 17 | 38,031,857 | T | -0.094 | 0.017 | 2.00E-09 |
| rs947474 | 10 | 6,390,450 | A | 0.122 | 0.023 | 2.80E-09 |
| rs1432296 | 2 | 61,068,167 | T | 0.122 | 0.018 | 3.60E-09 |
| rs592390 | 18 | 12,822,314 | T | 0.095 | 0.019 | 3.80E-09 |
| rs28411352 | 1 | 38,278,579 | T | 0.104 | 0.019 | 5.20E-09 |
| rs8133843 | 21 | 36,738,242 | A | 0.095 | 0.019 | 6.00E-09 |
| rs61828284 | 1 | 173,299,743 | T | -0.198 | 0.032 | 8.70E-09 |
| rs13330176 | 16 | 86,019,087 | A | 0.113 | 0.019 | 9.00E-09 |
| rs1893592 | 21 | 43,855,067 | A | 0.104 | 0.019 | 9.80E-09 |
| rs10985070 | 9 | 123,636,121 | A | -0.083 | 0.017 | 1.70E-08 |
| rs9310852 | 3 | 27,784,997 | A | -0.083 | 0.017 | 3.20E-08 |
| rs2233434 | 6 | 44,232,920 | A | -0.288 | 0.050 | 3.30E-08 |
| rs2234067 | 6 | 36,355,654 | A | -0.128 | 0.024 | 4.10E-08 |
| rs624988 | 1 | 117,263,790 | T | 0.086 | 0.014 | 4.60E-08 |
| rs4936059 | 11 | 128,502,496 | A | -0.094 | 0.017 | 4.60E-08 |
| rs73081554 | 3 | 58,302,935 | T | 0.166 | 0.031 | 4.70E-08 |
| rs144264189 | 6 | 26,311,416 | A | -0.301 | 0.051 | 6.00E-08 |
| rs73194058 | 21 | 34,764,288 | A | -0.128 | 0.024 | 7.10E-08 |
| rs773125 | 12 | 56,394,954 | A | 0.086 | 0.014 | 8.50E-08 |
| rs186735625 | 7 | 28,219,956 | T | -0.163 | 0.031 | 8.70E-08 |
| rs1633360 | 12 | 58,108,052 | T | 0.086 | 0.019 | 9.10E-08 |
| rs9826828 | 3 | 136,402,060 | A | 0.351 | 0.065 | 9.20E-08 |
| rs4657041 | 1 | 161,478,859 | T | 0.086 | 0.014 | 9.80E-08 |
| rs10175798 | 2 | 30,449,594 | A | 0.086 | 0.014 | 1.30E-07 |
| rs998731 | 8 | 81,095,395 | T | 0.086 | 0.019 | 1.30E-07 |
| rs6930468 | 6 | 426,268 | A | -0.083 | 0.017 | 1.60E-07 |
| rs187339910 | 1 | 2,523,878 | A | 0.095 | 0.019 | 1.80E-07 |
| rs2253125 | 3 | 128,329,793 | T | -0.174 | 0.031 | 1.90E-07 |
| rs1950897 | 14 | 68,760,141 | T | 0.086 | 0.014 | 2.50E-07 |
| rs6715284 | 2 | 202,154,397 | G | 0.131 | 0.028 | 2.90E-07 |
| rs71540792 | 7 | 17,216,929 | T | -0.128 | 0.030 | 2.90E-07 |
| rs12413578 | 10 | 9,049,253 | T | -0.186 | 0.038 | 3.30E-07 |
| rs4272 | 7 | 92,236,829 | A | -0.094 | 0.023 | 3.40E-07 |
| rs9262585 | 6 | 31,016,795 | T | -0.545 | 0.107 | 3.60E-07 |
| rs11075010 | 16 | 11,826,013 | T | 0.086 | 0.019 | 3.80E-07 |
| rs79893749 | 3 | 46,253,650 | T | -0.117 | 0.023 | 4.10E-07 |
| rs73000522 | 11 | 107,966,040 | T | -0.186 | 0.038 | 4.50E-07 |
| rs111480654 | 4 | 27,782,743 | T | -0.431 | 0.085 | 4.80E-07 |
| rs7224929 | 17 | 5,011,736 | A | 0.174 | 0.036 | 4.80E-07 |

**Table S8.** Associations between genetic risks and RA in different sex-stratified groups in test set.

| **Subgroups** | **Genetic risk** | **Cases/**  **Controls** | **Case-control analysis** | | **Incidence/**  **100,000 py** | **Prospective analysis** | |
| --- | --- | --- | --- | --- | --- | --- | --- |
|  |  |  | **OR (95%CI)** | ***P*** |  | **HR (95%CI)** | ***P*** |
| All | Low risk | 486/55,879 | **Ref** |  | 56.611 | **Ref** |  |
| All | Intermediate risk | 1,313/111,416 | 1.347 (1.213, 1.496) | 2.40E-08 | 70.394 | 1.237 (1.098, 1.393) | 4.56E-04 |
| All | High risk | 1,045/55,320 | 2.169 (1.946, 2.417) | 1.80E-44 | 108.183 | 1.910 (1.687, 2.161) | 1.31E-24 |
| Men | Low risk | 167/26,098 | **Ref** |  | 39.436 | **Ref** |  |
| Men | Intermediate risk | 417/51,695 | 1.258 (1.050, 1.507) | 0.012 | 51.083 | 1.294 (1.052, 1.593) | 0.015 |
| Men | High risk | 351/25,860 | 2.113 (1.755, 2.543) | 2.60E-15 | 82.092 | 2.076 (1.676, 2.572) | 2.37E-11 |
| Women | Low risk | 319/29,781 | **Ref** |  | 71.597 | **Ref** |  |
| Women | Intermediate risk | 896/59,721 | 1.395 (1.227, 1.587) | 3.90E-07 | 87.025 | 1.211 (1.047, 1.399) | 0.010 |
| Women | High risk | 694/29,460 | 2.197 (1.922, 2.511) | 8.97E-31 | 130.928 | 1.829 (1.571, 2.129) | 6.48E-15 |

Note: Defined by quintiles of PRS: low (the bottom quartile), intermediate (quintiles 2-3) and high (the top quintile). Associations were adjusted for age, sex, genotyped batch, assessment center, Townsend deprivation index and the first 10 principal components of ancestry. Abbreviations: CI, confidence interval; OR, odds ratio; HR, hazard ratio; PRS, polygenic risk score; Ref, reference; MHC, major histocompatibility complex.

**Table S9.** Associations between genetic risks (non-MHC) and RA in different sex-stratified groups in test set.

| **Subgroups** | **Genetic risk** | **Cases/**  **Controls** | **Case-control analysis** | | **Incidence/**  **100,000 py** | **Prospective analysis** | |
| --- | --- | --- | --- | --- | --- | --- | --- |
|  |  |  | **OR (95%CI)** | ***P*** |  | **HR (95%CI)** | ***P*** |
| All | Low risk | 1,045/55,320 | **Ref** |  | 58.093 | **Ref** |  |
| All | Intermediate risk | 1,313/111,416 | 1.336 (1.205, 1.481) | 3.54E-08 | 72.616 | 1.246 (1.108, 1.400) | 2.39E-04 |
| All | High risk | 486/55,879 | 1.997 (1.793, 2.225) | 4.17E-36 | 102.255 | 1.762 (1.557, 1.995) | 3.42E-19 |
| Men | Low risk | 167/26,040 | **Ref** |  | 39.840 | **Ref** |  |
| Men | Intermediate risk | 434/51,808 | 1.301 (1.088, 1.557) | 0.004 | 53.353 | 1.333 (1.086, 1.638) | 0.006 |
| Men | High risk | 334/25,805 | 2.013 (1.669, 2.426) | 2.20E-13 | 77.211 | 1.933 (1.557, 2.398) | 2.23E-09 |
| Women | Low risk | 336/29,822 | **Ref** |  | 73.955 | **Ref** |  |
| Women | Intermediate risk | 911/59,576 | 1.353 (1.193, 1.535) | 2.55E-06 | 89.283 | 1.204 (1.044, 1.388) | 0.011 |
| Women | High risk | 662/29,564 | 1.988 (1.741, 2.269) | 2.81E-24 | 123.977 | 1.680 (1.444, 1.955) | 1.94E-11 |

Note: Defined by quintiles of PRS: low (the bottom quartile), intermediate (quintiles 2-3) and high (the top quintile). Associations were adjusted for age, sex, genotyped batch, assessment center, Townsend deprivation index and the first 10 principal components of ancestry. Abbreviations: CI, confidence interval; OR, odds ratio; HR, hazard ratio; PRS, polygenic risk score; Ref, reference; MHC, major histocompatibility complex.

**Table S10.** Associations between healthy lifestyles and RA risks in test set.

| **Gender** | **Lifestyle** | **Cases/**  **Controls** | **Case-control analysis** | | **Incidence/**  **100,000 py** | **Prospective analysis** | |
| --- | --- | --- | --- | --- | --- | --- | --- |
|  |  |  | **OR (95%CI)** | ***P*** |  | **HR (95%CI)** | ***P*** |
| All | Favorable | 718/80,004 | **Ref** |  | 56.351 | **Ref** |  |
| All | Intermediate | 1,134/93,121 | 1.342 (1.221, 1.475) | 9.72E-10 | 73.545 | 1.290 (1.158, 1.438) | 4.07E-06 |
| All | Unfavorable | 992/49,490 | 2.152 (1.950, 2.374) | 8.41E-53 | 113.853 | 1.944 (1.735, 2.179) | 3.06E-30 |
| Men | Favorable | 209/35,825 | **Ref** |  | 39.576 | **Ref** |  |
| Men | Intermediate | 369/43,389 | 1.395 (1.176, 1.656) | 1.33E-04 | 50.933 | 1.236 (1.019, 1.498) | 0.031 |
| Men | Unfavorable | 357/24,439 | 2.257 (1.897, 2.684) | 3.85E-20 | 88.584 | 2.030 (1.671, 2.467) | 1.02E-12 |
| Women | Favorable | 509/44,179 | **Ref** |  | 69.898 | **Ref** |  |
| Women | Intermediate | 765/49,732 | 1.317 (1.175, 1.475) | 1.98E-06 | 93.159 | 1.314 (1.152, 1.498) | 4.51E-05 |
| Women | Unfavorable | 635/25,051 | 2.095 (1.859, 2.361) | 7.37E-34 | 138.329 | 1.888 (1.639, 2.174) | 1.10E-18 |

Note: Participants were divided into favorable (3 healthy lifestyle factors), intermediate (2 healthy lifestyle factors), or unfavorable (0 or 1 healthy lifestyle factor) according to the number of lifestyle factors. Associations were adjusted for age, sex, genotyped batch, assessment center, Townsend deprivation index and the first 10 principal components of ancestry. Abbreviations: CI, confidence interval; OR, odds ratio; HR, hazard ratio; PRS, polygenic risk score; Ref, reference.

**Table S11.** Sex-specific risks of RA according to genetic and lifestyle categories in the UKB cohort in test set (Case-control analysis).

| **Subgroups** | | **Men (N = 104,588)** | | |  | **Women (N = 120,871)** | | |
| --- | --- | --- | --- | --- | --- | --- | --- | --- |
|  |  | **Cases/**  **Controls** | **OR (95%CI)** | ***P*** |  | **Cases/**  **Controls** | **OR (95%CI)** | ***P*** |
| Low genetic risk | |  |  |  |  |  |  |  |
|  | Favorable | 30/8,909 | Ref |  |  | 91/11,024 | Ref |  |
|  | Intermediate | 68/10,982 | 1.752 (1.139, 2.696) | 0.011 |  | 128/12,415 | 1.231 (0.939, 1.613) | 0.132 |
|  | Unfavorable | 69/6,207 | 2.978 (1.936, 4.583) | 6.90E-07 |  | 100/6,342 | 1.817 (1.364, 2.420) | 4.44E-05 |
|  |  |  | *P* _trend_ = 8.61E-09 |  |  |  | *P* _trend_ = 1.06E-05 |  |
| Intermediate genetic risk | |  |  |  |  |  |  |  |
|  | Favorable | 105/17,971 | 1.732 (1.153, 2.601) | 0.008 |  | 249/22,311 | 1.345 (1.056, 1.712) | 0.016 |
|  | Intermediate | 160/21,585 | 2.092 (1.415, 3.093) | 2.18E-04 |  | 340/24,958 | 1.618 (1.282, 2.042) | 5.10E-05 |
|  | Unfavorable | 152/12,139 | 3.353 (2.262, 4.970) | 1.69E-09 |  | 307/12,452 | 2.832 (2.236, 3.587) | 6.11E-18 |
|  |  |  | *P* _trend_ = 1.25E-09 |  |  |  | *P* _trend_ = 5.54E-20 |  |
| High genetic risk | |  |  |  |  |  |  |  |
|  | Favorable | 74/8,945 | 2.431 (1.588, 3.720) | 4.31E-05 |  | 169/10,844 | 1.886 (1.459, 2.437) | 1.24E-06 |
|  | Intermediate | 141/10,822 | 3.719 (2.504, 5.524) | 7.57E-11 |  | 297/12,359 | 2.866 (2.262, 3.631) | 2.74E-18 |
|  | Unfavorable | 136/6,093 | 5.879 (3.949, 8.752) | 2.70E-18 |  | 228/6,257 | 4.175 (3.264, 5.339) | 4.95E-30 |
|  |  |  | *P* _trend_ = 1.55E-12 |  |  |  | *P* _trend_ = 4.74E-17 |  |

Note: Defined by quintiles of PRS: low (the bottom quartile), intermediate (quintiles 2-3) and high (the top quintile). Participants were divided into favorable (3 healthy lifestyle factors), intermediate (2 healthy lifestyle factors), or unfavorable (0 or 1 healthy lifestyle factor) according to the number of lifestyle factors. *P* _trend_ is P values for Cochran-Armitage trend test. Associations were adjusted for age, sex, genotyped batch, assessment center, Townsend deprivation index and the first 10 principal components of ancestry. Abbreviations: CI, confidence interval; OR, odds ratio; PRS, polygenic risk score; Ref, reference.

**Table S12.** Sex-specific risks of RA according to genetic and lifestyle categories in the UKB cohort in test set (Prospective analysis).

| **Subgroups** | | **Men (N = 104,581)** | | |  | **Women (N = 120,348)** | | |
| --- | --- | --- | --- | --- | --- | --- | --- | --- |
|  |  | **Incidence/**  **100,000 py** | **HR (95%CI)** | ***P*** |  | **Incidence/**  **100,000 py** | **HR (95%CI)** | ***P*** |
| Low genetic risk | |  |  |  |  |  |  |  |
|  | Favorable | 21.480 | Ref |  |  | 59.304 | Ref |  |
|  | Intermediate | 40.086 | 1.780 (1.091, 2.905) | 0.021 |  | 66.629 | 1.109 (0.825, 1.490) | 0.492 |
|  | Unfavorable | 63.877 | 2.692 (1.636, 4.429) | 9.67E-05 |  | 102.528 | 1.651 (1.207, 2.257) | 1.69E-03 |
|  |  |  | *P* _trend_ = 7.28E-08 |  |  |  | *P* _trend_ = 7.73E-04 |  |
| Intermediate genetic risk | |  |  |  |  |  |  |  |
|  | Favorable | 40.144 | 1.864 (1.177, 2.952) | 0.008 |  | 65.203 | 1.096 (0.840, 1.429) | 0.500 |
|  | Intermediate | 44.142 | 1.959 (1.252, 3.067) | 0.003 |  | 82.547 | 1.365 (1.060, 1.759) | 0.016 |
|  | Unfavorable | 79.462 | 3.353 (2.142, 5.248) | 1.20E-07 |  | 134.610 | 2.159 (1.664, 2.802) | 7.17E-09 |
|  |  |  | *P* _trend_ = 1.12E-06 |  |  |  | *P* _trend_ = 1.78E-12 |  |
| High genetic risk | |  |  |  |  |  |  |  |
|  | Favorable | 56.331 | 2.590 (1.603, 4.185) | 1.01E-04 |  | 90.201 | 1.522 (1.146, 2.023) | 0.004 |
|  | Intermediate | 75.294 | 3.376 (2.144, 5.315) | 1.49E-07 |  | 140.683 | 2.343 (1.809, 3.034) | 1.07E-10 |
|  | Unfavorable | 131.530 | 5.480 (3.476, 8.640) | 2.41E-13 |  | 181.413 | 2.904 (2.202, 3.830) | 4.32E-14 |
|  |  |  | *P* _trend_ = 8.14E-08 |  |  |  | *P* _trend_ = 8.30E-09 |  |

Note: Defined by quintiles of PRS: low (the bottom quartile), intermediate (quintiles 2-3) and high (the top quintile). Participants were divided into favorable (3 healthy lifestyle factors), intermediate (2 healthy lifestyle factors), or unfavorable (0 or 1 healthy lifestyle factor) according to the number of lifestyle factors. *P* _trend_ is P values for Cochran-Armitage trend test. Associations were adjusted for age, sex, genotyped batch, assessment center, Townsend deprivation index and the first 10 principal components of ancestry. Abbreviations: CI, confidence interval; HR, hazard ratio; PRS, polygenic risk score; Ref, reference.

**Table S13.** RA risks according to genetic (PRS _non-MHC_) and lifestyle categories in test set (Case-control analysis).

| **Subgroups** | | **All** | | **Men** | | **Women** | |
| --- | --- | --- | --- | --- | --- | --- | --- |
|  |  | **OR (95%CI)** | ***P*** | **OR (95%CI)** | ***P*** | **OR (95%CI)** | ***P*** |
| Low genetic risk | |  |  |  |  |  |  |
|  | Favorable | **Ref** |  | **Ref** |  | **Ref** |  |
|  | Intermediate | 1.453 (1.157, 1.826) | 1.32E-03 | 1.521 (0.998, 2.318) | 0.051 | 1.424 (1.085, 1.869) | 0.011 |
|  | Unfavorable | 2.440 (1.933, 3.082) | 6.60E-14 | 2.834 (1.869, 4.296) | 9.35E-07 | 2.256 (1.698, 2.998) | 2.01E-08 |
|  |  | *P* _trend_ = 1.51E-11 |  | *P* _trend_ = 1.42E-08 |  | *P* _trend_ = 1.47E-09 |  |
| Intermediate genetic risk | |  |  |  |  |  |  |
|  | Favorable | 1.547 (1.256, 1.905) | 4.09E-05 | 1.623 (1.098, 2.401) | 0.015 | 1.516 (1.185, 1.940) | 9.23E-04 |
|  | Intermediate | 1.813 (1.482, 2.218) | 7.01E-09 | 1.937 (1.331, 2.820) | 5.59E-04 | 1.759 (1.385, 2.235) | 3.62E-06 |
|  | Unfavorable | 3.150 (2.571, 3.859) | 1.69E-28 | 3.322 (2.282, 4.838) | 3.81E-10 | 3.073 (2.412, 3.914) | 1.01E-19 |
|  |  | *P* _trend_ = 3.13E-26 |  | *P* _trend_ = 1.50E-11 |  | *P* _trend_ = 1.23E-18 |  |
| High genetic risk | |  |  |  |  |  |  |
|  | Favorable | 2.020 (1.618, 2.523) | 5.55E-10 | 2.101 (1.386, 3.186) | 4.70E-04 | 1.986 (1.526, 2.583) | 3.18E-07 |
|  | Intermediate | 3.125 (2.546, 3.836) | 1.13E-27 | 3.491 (2.387, 5.106) | 1.14E-10 | 2.969 (2.327, 3.789) | 2.11E-18 |
|  | Unfavorable | 4.392 (3.552, 5.430) | 1.61E-42 | 4.819 (3.272, 7.099) | 1.73E-15 | 4.188 (3.245, 5.405) | 3.61E-28 |
|  |  | *P* _trend_ = 1.39E-26 |  | *P* _trend_ = 2.35E-10 |  | *P* _trend_ = 1.65E-14 |  |

Note: Defined by quintiles of PRS: low (the bottom quartile), intermediate (quintiles 2-3) and high (the top quintile). Participants were divided into favorable (3 healthy lifestyle factors), intermediate (2 healthy lifestyle factors), or unfavorable (0 or 1 healthy lifestyle factor) according to the number of lifestyle factors. *P* _trend_ is P values for Cochran-Armitage trend test. Associations were adjusted for age, sex, genotyped batch, assessment center, Townsend deprivation index and the first 10 principal components of ancestry. Abbreviations: CI, confidence interval; OR, odds ratio; HR, hazard ratio; PRS, polygenic risk score; Ref, reference.

**Table S14.** RA risks according to genetic (PRS _non-MHC_) and lifestyle categories in test set (Prospective analysis).

| **Subgroups** | | **All** | | **Men** | | **Women** | |
| --- | --- | --- | --- | --- | --- | --- | --- |
|  |  | **HR (95%CI)** | ***P*** | **HR (95%CI)** | ***P*** | **HR (95%CI)** | ***P*** |
| Low genetic risk | |  |  |  |  |  |  |
|  | Favorable | **Ref** |  | **Ref** |  | **Ref** |  |
|  | Intermediate | 1.453 (1.157, 1.826) | 1.32E-03 | 1.521 (0.998, 2.318) | 0.051 | 1.424 (1.085, 1.869) | 0.011 |
|  | Unfavorable | 2.440 (1.933, 3.082) | 6.60E-14 | 2.834 (1.869, 4.296) | 9.35E-07 | 2.256 (1.698, 2.998) | 2.01E-08 |
|  |  | *P* _trend_ = 1.03E-10 |  | *P* _trend_ = 3.98E-05 |  | *P* _trend_ = 1.24E-07 |  |
| Intermediate genetic risk | |  |  |  |  |  |  |
|  | Favorable | 1.547 (1.256, 1.905) | 4.09E-05 | 1.623 (1.098, 2.401) | 0.015 | 1.516 (1.185, 1.940) | 9.23E-04 |
|  | Intermediate | 1.813 (1.482, 2.218) | 7.01E-09 | 1.937 (1.331, 2.820) | 5.59E-04 | 1.759 (1.385, 2.235) | 3.62E-06 |
|  | Unfavorable | 3.150 (2.571, 3.859) | 1.69E-28 | 3.322 (2.282, 4.838) | 3.81E-10 | 3.073 (2.412, 3.914) | 1.01E-19 |
|  |  | *P* _trend_ = 6.19E-15 |  | *P* _trend_ = 7.99E-08 |  | *P* _trend_ = 1.37E-09 |  |
| High genetic risk | |  |  |  |  |  |  |
|  | Favorable | 2.020 (1.618, 2.523) | 5.55E-10 | 2.101 (1.386, 3.186) | 4.70E-04 | 1.986 (1.526, 2.583) | 3.18E-07 |
|  | Intermediate | 3.125 (2.546, 3.836) | 1.13E-27 | 3.491 (2.387, 5.106) | 1.14E-10 | 2.969 (2.327, 3.789) | 2.11E-18 |
|  | Unfavorable | 4.392 (3.552, 5.430) | 1.61E-42 | 4.819 (3.272, 7.099) | 1.73E-15 | 4.188 (3.245, 5.405) | 3.61E-28 |
|  |  | *P* _trend_ = 6.80E-13 |  | *P* _trend_ = 5.02E-07 |  | *P* _trend_ = 3.70E-08 |  |

Note: Defined by quintiles of PRS: low (the bottom quartile), intermediate (quintiles 2-3) and high (the top quintile). Participants were divided into favorable (3 healthy lifestyle factors), intermediate (2 healthy lifestyle factors), or unfavorable (0 or 1 healthy lifestyle factor) according to the number of lifestyle factors. *P* _trend_ is P values for Cochran-Armitage trend test. Associations were adjusted for age, sex, genotyped batch, assessment center, Townsend deprivation index and the first 10 principal components of ancestry. Abbreviations: CI, confidence interval; OR, odds ratio; HR, hazard ratio; PRS, polygenic risk score; Ref, reference.

**Table S15.** RA risks according to genetic (PRS) and lifestyle categories in test set (Case-control analysis) by integrating five lifestyles.

| **Subgroups** | | **All** | | **Men** | | **Women** | |
| --- | --- | --- | --- | --- | --- | --- | --- |
|  |  | **OR (95%CI)** | ***P*** | **OR (95%CI)** | ***P*** | **OR (95%CI)** | ***P*** |
| Low genetic risk | |  |  |  |  |  |  |
|  | Favorable | **Ref** |  | **Ref** |  | **Ref** |  |
|  | Intermediate | 0.982 (0.763, 1.264) | 0.888 | 0.87 (0.53, 1.429) | 0.583 | 1.024 (0.764, 1.374) | 0.872 |
|  | Unfavorable | 1.565 (1.168, 2.096) | 0.003 | 1.811 (1.073, 3.057) | 0.026 | 1.329 (0.919, 1.921) | 0.131 |
|  |  | *P* _trend_ = 0.008 |  | *P* _trend_ = 0.157 |  | *P* _trend_ = 3.25E-04 |  |
| Intermediate genetic risk | |  |  |  |  |  |  |
|  | Favorable | 1.159 (0.886, 1.516) | 0.282 | 0.853 (0.482, 1.508) | 0.584 | 1.256 (0.925, 1.705) | 0.144 |
|  | Intermediate | 1.353 (1.069, 1.711) | 5.77E-06 | 1.289 (0.808, 2.056) | 0.287 | 1.365 (1.04, 1.792) | 0.025 |
|  | Unfavorable | 2.196 (1.708, 2.825) | 8.96E-16 | 1.874 (1.15, 3.054) | 0.012 | 2.352 (1.751, 3.16) | 1.37E-08 |
|  |  | *P* _trend_ = 4.53E-10 |  | *P* _trend_ = 3.03E-06 |  | *P* _trend_ = 9.42E-09 |  |
| High genetic risk | |  |  |  |  |  |  |
|  | Favorable | 1.615 (1.213, 2.152) | 1.05E-03 | 1.458 (0.812, 2.618) | 0.207 | 1.665 (1.198, 2.313) | 2.40E-03 |
|  | Intermediate | 2.283 (1.801, 2.895) | 9.06E-12 | 2.112 (1.319, 3.381) | 0.002 | 2.333 (1.772, 3.072) | 1.55E-09 |
|  | Unfavorable | 3.34 (2.575, 4.331) | 9.47E-20 | 3.307 (2.019, 5.416) | 2.02E-06 | 3.244 (2.378, 4.426) | 1.13E-13 |
|  |  | *P* _trend_ = 2.05E-09 |  | *P* _trend_ = 1.25E-06 |  | *P* _trend_ = 6.85E-07 |  |

Note: Defined by quintiles of PRS: low (the bottom quartile), intermediate (quintiles 2-3) and high (the top quintile). Participants were divided into favorable (3 healthy lifestyle factors), intermediate (2 healthy lifestyle factors), or unfavorable (0 or 1 healthy lifestyle factor) according to the number of lifestyle factors. *P* _trend_ is P values for Cochran-Armitage trend test. Associations were adjusted for age, sex, genotyped batch, assessment center, Townsend deprivation index and the first 10 principal components of ancestry. Abbreviations: CI, confidence interval; OR, odds ratio; HR, hazard ratio; PRS, polygenic risk score; Ref, reference.

**Table S16.** RA risks according to genetic (PRS) and lifestyle categories in test set (Prospective analysis) by integrating five lifestyles.

| **Subgroups** | | **All** | | **Men** | | **Women** | |
| --- | --- | --- | --- | --- | --- | --- | --- |
|  |  | **HR (95%CI)** | ***P*** | **HR (95%CI)** | ***P*** | **HR (95%CI)** | ***P*** |
| Low genetic risk | |  |  |  |  |  |  |
|  | Favorable | **Ref** |  | **Ref** |  | **Ref** |  |
|  | Intermediate | 0.878 (0.67, 1.151) | 0.346 | 0.865 (0.496, 1.509) | 0.610 | 0.896 (0.657, 1.222) | 0.488 |
|  | Unfavorable | 1.225 (0.885, 1.696) | 0.221 | 1.516 (0.832, 2.764) | 0.174 | 1.072 (0.714, 1.609) | 0.737 |
|  |  | *P* _trend_ = 0.426 |  | *P* _trend_ = 0.864 |  | *P* _trend_ = 0.028 |  |
| Intermediate genetic risk | |  |  |  |  |  |  |
|  | Favorable | 0.903 (0.672, 1.214) | 0.500 | 0.741 (0.384, 1.428) | 0.371 | 0.948 (0.68, 1.321) | 0.754 |
|  | Intermediate | 1.111 (0.865, 1.429) | 0.410 | 1.238 (0.733, 2.092) | 0.424 | 1.071 (0.804, 1.426) | 0.641 |
|  | Unfavorable | 1.769 (1.347, 2.322) | 3.99E-05 | 1.913 (1.107, 3.304) | 0.020 | 1.724 (1.253, 2.37) | 8.13E-04 |
|  |  | *P* _trend_ = 1.77E-08 |  | *P* _trend_ = 4.99E-06 |  | *P* _trend_ = 1.57E-06 |  |
| High genetic risk | |  |  |  |  |  |  |
|  | Favorable | 1.243 (0.903, 1.711) | 0.183 | 1.183 (0.596, 2.347) | 0.631 | 1.261 (0.878, 1.81) | 0.209 |
|  | Intermediate | 1.798 (1.395, 2.318) | 6.03E-06 | 2.011 (1.186, 3.411) | 0.010 | 1.724 (1.288, 2.306) | 2.48E-04 |
|  | Unfavorable | 2.534 (1.908, 3.366) | 1.38E-10 | 2.981 (1.708, 5.202) | 1.21E-04 | 2.322 (1.653, 3.26) | 1.15E-06 |
|  |  | *P* _trend_ = 8.23E-07 |  | *P* _trend_ = 1.39E-04 |  | *P* _trend_ = 2.61E-05 |  |

Note: Defined by quintiles of PRS: low (the bottom quartile), intermediate (quintiles 2-3) and high (the top quintile). Participants were divided into favorable (3 healthy lifestyle factors), intermediate (2 healthy lifestyle factors), or unfavorable (0 or 1 healthy lifestyle factor) according to the number of lifestyle factors. *P* _trend_ is P values for Cochran-Armitage trend test. Associations were adjusted for age, sex, genotyped batch, assessment center, Townsend deprivation index and the first 10 principal components of ancestry. Abbreviations: CI, confidence interval; OR, odds ratio; HR, hazard ratio; PRS, polygenic risk score; Ref, reference.

**Table S17.** RA risks according to genetic (PRS) and lifestyle categories in test set (Case-control analysis) without excluding confounding disease.

| **Subgroups** | | **All** | | **Men** | | **Women** | |
| --- | --- | --- | --- | --- | --- | --- | --- |
|  |  | **OR (95%CI)** | ***P*** | **OR (95%CI)** | ***P*** | **OR (95%CI)** | ***P*** |
| Low genetic risk | |  |  |  |  |  |  |
|  | Favorable | **Ref** |  | **Ref** |  | **Ref** |  |
|  | Intermediate | 1.489 (1.196, 1.853) | 3.70E-04 | 1.795 (1.193, 2.701) | 0.005 | 1.369 (1.055, 1.777) | 0.018 |
|  | Unfavorable | 2.583 (2.075, 3.216) | 2.18E-17 | 2.977 (1.981, 4.475) | 1.54E-07 | 2.429 (1.870, 3.155) | 2.92E-11 |
|  |  | *P* _trend_ = 1.04E-21 |  | *P* _trend_ = 3.74E-10 |  | *P* _trend_ = 6.40E-14 |  |
| Intermediate genetic risk | |  |  |  |  |  |  |
|  | Favorable | 1.489 (1.215, 1.824) | 1.25E-04 | 1.688 (1.144, 2.490) | 0.008 | 1.417 (1.116, 1.799) | 0.004 |
|  | Intermediate | 1.947 (1.604, 2.364) | 1.62E-11 | 2.530 (1.752, 3.654) | 7.48E-07 | 1.724 (1.371, 2.168) | 3.20E-06 |
|  | Unfavorable | 3.260 (2.682, 3.962) | 1.78E-32 | 3.505 (2.416, 5.084) | 3.86E-11 | 3.179 (2.526, 4.000) | 6.22E-23 |
|  |  | *P* _trend_ = 1.22E-38 |  | *P* _trend_ = 2.86E-13 |  | *P* _trend_ = 7.41E-29 |  |
| High genetic risk | |  |  |  |  |  |  |
|  | Favorable | 2.162 (1.746, 2.676) | 1.45E-12 | 2.754 (1.848, 4.104) | 6.42E-07 | 1.943 (1.507, 2.504) | 2.97E-07 |
|  | Intermediate | 3.131 (2.567, 3.819) | 1.87E-29 | 3.486 (2.387, 5.092) | 1.05E-10 | 2.999 (2.374, 3.788) | 3.06E-20 |
|  | Unfavorable | 4.763 (3.891, 5.830) | 9.08E-52 | 5.271 (3.601, 7.715) | 1.21E-17 | 4.576 (3.601, 5.814) | 1.46E-35 |
|  |  | *P* _trend_ = 3.71E-28 |  | *P* _trend_ = 4.46E-09 |  | *P* _trend_ = 4.00E-22 |  |

Note: Defined by quintiles of PRS: low (the bottom quartile), intermediate (quintiles 2-3) and high (the top quintile). Participants were divided into favorable (3 healthy lifestyle factors), intermediate (2 healthy lifestyle factors), or unfavorable (0 or 1 healthy lifestyle factor) according to the number of lifestyle factors. *P* _trend_ is P values for Cochran-Armitage trend test. Associations were adjusted for age, sex, genotyped batch, assessment center, Townsend deprivation index and the first 10 principal components of ancestry. Abbreviations: CI, confidence interval; OR, odds ratio; HR, hazard ratio; PRS, polygenic risk score; Ref, reference.

**Table S18.** RA risks according to genetic (PRS) and lifestyle categories in test set (Prospective analysis) without excluding confounding disease.

| **Subgroups** | | **All** | | **Men** | | **Women** | |
| --- | --- | --- | --- | --- | --- | --- | --- |
|  |  | **HR (95%CI)** | ***P*** | **HR (95%CI)** | ***P*** | **HR (95%CI)** | ***P*** |
| Low genetic risk | |  |  |  |  |  |  |
|  | Favorable | **Ref** |  | **Ref** |  | **Ref** |  |
|  | Intermediate | 1.358 (1.100, 1.677) | 0.004 | 1.596 (1.067, 2.386) | 0.023 | 1.274 (0.995, 1.633) | 0.055 |
|  | Unfavorable | 2.242 (1.814, 2.772) | 8.70E-14 | 2.566 (1.715, 3.840) | 4.52E-06 | 2.134 (1.661, 2.742) | 3.10E-09 |
|  |  | *P* _trend_ = 6.09E-17 |  | *P* _trend_ = 7.21E-08 |  | *P* _trend_ = 1.89E-11 |  |
| Intermediate genetic risk | |  |  |  |  |  |  |
|  | Favorable | 1.355 (1.115, 1.646) | 0.002 | 1.718 (1.180, 2.501) | 0.005 | 1.232 (0.981, 1.548) | 0.073 |
|  | Intermediate | 1.797 (1.494, 2.161) | 5.08E-10 | 2.406 (1.684, 3.438) | 1.41E-06 | 1.580 (1.271, 1.962) | 3.65E-05 |
|  | Unfavorable | 2.695 (2.234, 3.252) | 3.92E-25 | 3.158 (2.198, 4.539) | 5.15E-10 | 2.537 (2.035, 3.161) | 1.14E-16 |
|  |  | *P* _trend_ = 8.81E-30 |  | *P* _trend_ = 5.81E-10 |  | *P* _trend_ = 6.39E-23 |  |
| High genetic risk | |  |  |  |  |  |  |
|  | Favorable | 1.822 (1.481, 2.242) | 1.41E-08 | 2.391 (1.615, 3.540) | 1.33E-05 | 1.621 (1.268, 2.073) | 1.15E-04 |
|  | Intermediate | 2.702 (2.234, 3.269) | 1.40E-24 | 3.169 (2.191, 4.585) | 9.24E-10 | 2.535 (2.029, 3.168) | 2.70E-16 |
|  | Unfavorable | 3.775 (3.105, 4.591) | 1.98E-40 | 4.563 (3.142, 6.627) | 1.56E-15 | 3.500 (2.778, 4.409) | 2.28E-26 |
|  |  | *P* _trend_ = 3.73E-23 |  | *P* _trend_ = 3.72E-08 |  | *P* _trend_ = 1.24E-17 |  |

Note: Defined by quintiles of PRS: low (the bottom quartile), intermediate (quintiles 2-3) and high (the top quintile). Participants were divided into favorable (3 healthy lifestyle factors), intermediate (2 healthy lifestyle factors), or unfavorable (0 or 1 healthy lifestyle factor) according to the number of lifestyle factors. *P* _trend_ is P values for Cochran-Armitage trend test. Associations were adjusted for age, sex, genotyped batch, assessment center, Townsend deprivation index and the first 10 principal components of ancestry. Abbreviations: CI, confidence interval; OR, odds ratio; HR, hazard ratio; PRS, polygenic risk score; Ref, reference.

**Table S19.** RA risks according to genetic (PRS) and lifestyle categories in test set (60%) (Case-control analysis).

| **Subgroups** | | **All** | | **Men** | | **Women** | |
| --- | --- | --- | --- | --- | --- | --- | --- |
|  |  | **OR (95%CI)** | ***P*** | **OR (95%CI)** | ***P*** | **OR (95%CI)** | ***P*** |
| Low genetic risk | |  |  |  |  |  |  |
|  | Favorable | **Ref** |  | **Ref** |  | **Ref** |  |
|  | Intermediate | 1.442 (1.107, 1.878) | 0.007 | 2.041 (1.202, 3.466) | 0.008 | 1.270 (0.933, 1.729) | 0.129 |
|  | Unfavorable | 2.061 (1.561, 2.723) | 3.48E-07 | 3.049 (1.776, 5.234) | 5.27E-05 | 1.770 (1.271, 2.465) | 7.29E-04 |
|  |  | *P* _trend_ = 4.67E-08 |  | *P* _trend_ = 4.06E-06 |  | *P* _trend_ = 2.08E-04 |  |
| Intermediate genetic risk | |  |  |  |  |  |  |
|  | Favorable | 1.440 (1.129, 1.836) | 0.003 | 2.211 (1.343, 3.640) | 0.002 | 1.226 (0.926, 1.623) | 0.156 |
|  | Intermediate | 1.781 (1.410, 2.250) | 1.26E-06 | 2.505 (1.543, 4.069) | 2.06E-04 | 1.577 (1.206, 2.062) | 8.67E-04 |
|  | Unfavorable | 3.070 (2.424, 3.888) | 1.28E-20 | 3.997 (2.454, 6.510) | 2.58E-08 | 2.823 (2.151, 3.705) | 7.41E-14 |
|  |  | *P* _trend_ = 1.12E-21 |  | *P* _trend_ = 1.29E-06 |  | *P* _trend_ = 4.58E-18 |  |
| High genetic risk | |  |  |  |  |  |  |
|  | Favorable | 2.115 (1.639, 2.730) | 8.64E-09 | 3.165 (1.889, 5.305) | 1.22E-05 | 1.819 (1.352, 2.446) | 7.57E-05 |
|  | Intermediate | 3.275 (2.585, 4.147) | 7.65E-23 | 4.551 (2.790, 7.422) | 1.26E-09 | 2.915 (2.222, 3.825) | 1.16E-14 |
|  | Unfavorable | 4.945 (3.883, 6.296) | 1.87E-38 | 6.963 (4.256, 11.390) | 1.10E-14 | 4.357 (3.291, 5.769) | 9.04E-25 |
|  |  | *P* _trend_ = 9.39E-22 |  | *P* _trend_ = 1.04E-08 |  | *P* _trend_ = 1.66E-15 |  |

Note: Defined by quintiles of PRS: low (the bottom quartile), intermediate (quintiles 2-3) and high (the top quintile). Participants were divided into favorable (3 healthy lifestyle factors), intermediate (2 healthy lifestyle factors), or unfavorable (0 or 1 healthy lifestyle factor) according to the number of lifestyle factors. *P* _trend_ is P values for Cochran-Armitage trend test. Associations were adjusted for age, sex, genotyped batch, assessment center, Townsend deprivation index and the first 10 principal components of ancestry. Abbreviations: CI, confidence interval; OR, odds ratio; HR, hazard ratio; PRS, polygenic risk score; Ref, reference.

**Table S20.** RA risks according to genetic (PRS) and lifestyle categories in test set (60%) (Prospective analysis).

| **Subgroups** | | **All** | | **Men** | | **Women** | |
| --- | --- | --- | --- | --- | --- | --- | --- |
|  |  | **HR (95%CI)** | ***P*** | **HR (95%CI)** | ***P*** | **HR (95%CI)** | ***P*** |
| Low genetic risk | |  |  |  |  |  |  |
|  | Favorable | **Ref** |  | **Ref** |  | **Ref** |  |
|  | Intermediate | 1.421 (1.057, 1.910) | 0.020 | 2.444 (1.310, 4.562) | 0.005 | 1.180 (0.837, 1.662) | 0.345 |
|  | Unfavorable | 2.003 (1.466, 2.735) | 1.26E-05 | 3.239 (1.698, 6.176) | 3.59E-04 | 1.721 (1.196, 2.475) | 0.003 |
|  |  | *P* _trend_ = 2.35E-06 |  | *P* _trend_ = 6.00E-05 |  | *P* _trend_ = 1.25E-03 |  |
| Intermediate genetic risk | |  |  |  |  |  |  |
|  | Favorable | 1.342 (1.020, 1.764) | 0.035 | 2.683 (1.486, 4.847) | 0.001 | 1.035 (0.755, 1.419) | 0.828 |
|  | Intermediate | 1.602 (1.231, 2.084) | 4.54E-04 | 2.537 (1.414, 4.551) | 0.002 | 1.389 (1.032, 1.870) | 0.030 |
|  | Unfavorable | 2.688 (2.058, 3.509) | 3.77E-13 | 4.495 (2.509, 8.056) | 4.41E-07 | 2.265 (1.670, 3.073) | 1.48E-07 |
|  |  | *P* _trend_ = 3.24E-14 |  | *P* _trend_ = 1.48E-04 |  | *P* _trend_ = 4.89E-12 |  |
| High genetic risk | |  |  |  |  |  |  |
|  | Favorable | 1.983 (1.488, 2.643) | 3.00E-06 | 3.833 (2.085, 7.047) | 1.53E-05 | 1.555 (1.114, 2.170) | 0.009 |
|  | Intermediate | 2.925 (2.241, 3.818) | 2.80E-15 | 4.513 (2.502, 8.138) | 5.47E-07 | 2.568 (1.901, 3.470) | 8.06E-10 |
|  | Unfavorable | 3.898 (2.956, 5.140) | 5.26E-22 | 7.157 (3.962, 12.927) | 6.85E-11 | 3.100 (2.248, 4.275) | 5.25E-12 |
|  |  | *P* _trend_ = 5.42E-11 |  | *P* _trend_ = 4.41E-05 |  | *P* _trend_ = 9.09E-08 |  |

Note: Defined by quintiles of PRS: low (the bottom quartile), intermediate (quintiles 2-3) and high (the top quintile). Participants were divided into favorable (3 healthy lifestyle factors), intermediate (2 healthy lifestyle factors), or unfavorable (0 or 1 healthy lifestyle factor) according to the number of lifestyle factors. *P* _trend_ is P values for Cochran-Armitage trend test. Associations were adjusted for age, sex, genotyped batch, assessment center, Townsend deprivation index and the first 10 principal components of ancestry. Abbreviations: CI, confidence interval; OR, odds ratio; HR, hazard ratio; PRS, polygenic risk score; Ref, reference.

**Table S21.** RA risks according to genetic (PRS) and lifestyle categories after redefining the appropriate BMI as 18.5~25 (Case-control analysis).

| **Subgroups** | | **All** | | **Men** | | **Women** | |
| --- | --- | --- | --- | --- | --- | --- | --- |
|  |  | **OR (95%CI)** | ***P*** | **OR (95%CI)** | ***P*** | **OR (95%CI)** | ***P*** |
| Low genetic risk | |  |  |  |  |  |  |
|  | Favorable | **Ref** |  | **Ref** |  | **Ref** |  |
|  | Intermediate | 1.108 (0.764, 1.605) | 0.590 | 1.113 (0.506, 2.447) | 0.790 | 1.131 (0.741, 1.725) | 0.568 |
|  | Unfavorable | 2.028 (1.434, 2.869) | 6.46E-05 | 2.707 (1.307, 5.607) | 0.007 | 1.764 (1.180, 2.638) | 0.006 |
|  |  | *P* _trend_ = 8.74E-08 |  | *P* _trend_ = 2.84E-06 |  | *P* _trend_ = 1.73E-04 |  |
| Intermediate genetic risk | |  |  |  |  |  |  |
|  | Favorable | 1.309 (0.904, 1.895) | 0.154 | 1.134 (0.492, 2.612) | 0.768 | 1.352 (0.895, 2.044) | 0.152 |
|  | Intermediate | 1.608 (1.150, 2.250) | 0.006 | 1.934 (0.939, 3.986) | 0.074 | 1.522 (1.040, 2.226) | 0.030 |
|  | Unfavorable | 2.424 (1.744, 3.369) | 1.36E-07 | 2.708 (1.329, 5.516) | 0.006 | 2.370 (1.632, 3.441) | 5.82E-06 |
|  |  | *P* _trend_ = 8.13E-12 |  | *P* _trend_ = 1.92E-06 |  | *P* _trend_ = 1.08E-09 |  |
| High genetic risk | |  |  |  |  |  |  |
|  | Favorable | 1.905 (1.290, 2.813) | 1.19E-03 | 2.304 (0.999, 5.314) | 0.050 | 1.802 (1.159, 2.801) | 0.009 |
|  | Intermediate | 2.617 (1.861, 3.679) | 3.11E-08 | 2.652 (1.272, 5.527) | 0.009 | 2.658 (1.808, 3.908) | 6.64E-07 |
|  | Unfavorable | 4.015 (2.880, 5.598) | 2.46E-16 | 4.701 (2.302, 9.602) | 2.16E-05 | 3.810 (2.610, 5.562) | 4.24E-12 |
|  |  | *P* _trend_ = 6.22E-12 |  | *P* _trend_ = 1.81E-06 |  | *P* _trend_ = 2.20E-09 |  |

Note: Defined by quintiles of PRS: low (the bottom quartile), intermediate (quintiles 2-3) and high (the top quintile). Participants were divided into favorable (3 healthy lifestyle factors), intermediate (2 healthy lifestyle factors), or unfavorable (0 or 1 healthy lifestyle factor) according to the number of lifestyle factors. *P* _trend_ is P values for Cochran-Armitage trend test. Associations were adjusted for age, sex, genotyped batch, assessment center, Townsend deprivation index and the first 10 principal components of ancestry. Abbreviations: CI, confidence interval; OR, odds ratio; HR, hazard ratio; PRS, polygenic risk score; Ref, reference.

**Table S22.** RA risks according to genetic (PRS) and lifestyle categories after redefining the appropriate BMI as 18.5~25 (Prospective analysis).

| **Subgroups** | | **All** | | **Men** | | **Women** | |
| --- | --- | --- | --- | --- | --- | --- | --- |
|  |  | **HR (95%CI)** | ***P*** | **HR (95%CI)** | ***P*** | **HR (95%CI)** | ***P*** |
| Low genetic risk | |  |  |  |  |  |  |
|  | Favorable | **Ref** |  | **Ref** |  | **Ref** |  |
|  | Intermediate | 1.188 (0.835, 1.690) | 0.337 | 1.191 (0.546, 2.598) | 0.660 | 1.229 (0.827, 1.826) | 0.307 |
|  | Unfavorable | 1.755 (1.253, 2.458) | 1.06E-03 | 2.209 (1.061, 4.600) | 0.034 | 1.654 (1.126, 2.430) | 0.010 |
|  |  | *P* _trend_ = 4.15E-05 |  | *P* _trend_ = 5.39E-04 |  | *P* _trend_ = 7.19E-04 |  |
| Intermediate genetic risk | |  |  |  |  |  |  |
|  | Favorable | 1.189 (0.830, 1.703) | 0.346 | 1.254 (0.552, 2.846) | 0.589 | 1.171 (0.785, 1.747) | 0.439 |
|  | Intermediate | 1.472 (1.065, 2.034) | 0.019 | 1.887 (0.917, 3.884) | 0.085 | 1.381 (0.960, 1.987) | 0.082 |
|  | Unfavorable | 2.232 (1.626, 3.064) | 6.84E-07 | 2.924 (1.439, 5.940) | 0.003 | 2.047 (1.433, 2.926) | 8.35E-05 |
|  |  | *P* _trend_ = 1.98E-12 |  | *P* _trend_ = 1.81E-07 |  | *P* _trend_ = 3.24E-09 |  |
| High genetic risk | |  |  |  |  |  |  |
|  | Favorable | 1.870 (1.287, 2.716) | 1.02E-03 | 2.620 (1.161, 5.916) | 0.020 | 1.695 (1.111, 2.585) | 0.014 |
|  | Intermediate | 2.285 (1.643, 3.178) | 9.00E-07 | 2.771 (1.333, 5.759) | 0.006 | 2.189 (1.509, 3.174) | 3.60E-05 |
|  | Unfavorable | 3.453 (2.505, 4.759) | 3.74E-14 | 4.695 (2.303, 9.571) | 2.08E-05 | 3.083 (2.143, 4.436) | 1.30E-09 |
|  |  | *P* _trend_ = 2.21E-09 |  | *P* _trend_ = 2.01E-05 |  | *P* _trend_ = 3.43E-07 |  |

Note: Defined by quintiles of PRS: low (the bottom quartile), intermediate (quintiles 2-3) and high (the top quintile). Participants were divided into favorable (3 healthy lifestyle factors), intermediate (2 healthy lifestyle factors), or unfavorable (0 or 1 healthy lifestyle factor) according to the number of lifestyle factors. *P* _trend_ is P values for Cochran-Armitage trend test. Associations were adjusted for age, sex, genotyped batch, assessment center, Townsend deprivation index and the first 10 principal components of ancestry. Abbreviations: CI, confidence interval; OR, odds ratio; HR, hazard ratio; PRS, polygenic risk score; Ref, reference. **Table S23.** RA risks according to genetic (PRS) and lifestyle categories after redefining smoking variable (Case-control analysis).

| **Subgroups** | | **All** | | **Men** | | **Women** | |
| --- | --- | --- | --- | --- | --- | --- | --- |
|  |  | **OR (95%CI)** | ***P*** | **OR (95%CI)** | ***P*** | **OR (95%CI)** | ***P*** |
| Low genetic risk | |  |  |  |  |  |  |
|  | Favorable | **Ref** |  | **Ref** |  | **Ref** |  |
|  | Intermediate | 1.601 (1.266, 2.025) | 8.76E-05 | 1.59 (1.051, 2.404) | 0.028 | 1.604 (1.205, 2.134) | 1.21E-03 |
|  | Unfavorable | 2.149 (1.599, 2.887) | 3.82E-07 | 2.017 (1.2, 3.391) | 0.008 | 2.217 (1.548, 3.175) | 1.40E-05 |
|  |  | *P* _trend_ = 1.00E-08 |  | *P* _trend_ = 0.002 |  | *P* _trend_ = 9.50E-07 |  |
| Intermediate genetic risk | |  |  |  |  |  |  |
|  | Favorable | 1.406 (1.152, 1.716) | 7.93E-04 | 1.591 (1.124, 2.252) | 0.009 | 1.318 (1.033, 1.682) | 0.026 |
|  | Intermediate | 2.154 (1.768, 2.625) | 5.77E-06 | 2.114 (1.49, 3.001) | 2.76E-05 | 2.174 (1.711, 2.763) | 2.04E-10 |
|  | Unfavorable | 3.077 (2.458, 3.853) | 8.96E-16 | 2.802 (1.877, 4.181) | 4.60E-07 | 3.219 (2.453, 4.225) | 3.52E-17 |
|  |  | *P* _trend_ = 1.12E-21 |  | *P* _trend_ = 4.88E-05 |  | *P* _trend_ = 3.45E-19 |  |
| High genetic risk | |  |  |  |  |  |  |
|  | Favorable | 2.528 (2.064, 3.096) | 3.23E-19 | 2.595 (1.815, 3.71) | 1.71E-07 | 2.491 (1.947, 3.187) | 3.95E-13 |
|  | Intermediate | 3.541 (2.889, 4.339) | 3.71E-34 | 3.695 (2.589, 5.273) | 6.03E-13 | 3.462 (2.702, 4.437) | 1.01E-22 |
|  | Unfavorable | 5.326 (4.223, 6.716) | 2.61E-45 | 5.133 (3.413, 7.72) | 3.98E-15 | 5.426 (4.091, 7.196) | 7.81E-32 |
|  |  | *P* _trend_ = 1.57E-16 |  | *P* _trend_ = 3.91E-06 |  | *P* _trend_ = 3.86E-12 |  |

Note: Defined by quintiles of PRS: low (the bottom quartile), intermediate (quintiles 2-3) and high (the top quintile). Participants were divided into favorable (3 healthy lifestyle factors), intermediate (2 healthy lifestyle factors), or unfavorable (0 or 1 healthy lifestyle factor) according to the number of lifestyle factors. *P* _trend_ is P values for Cochran-Armitage trend test. Associations were adjusted for age, sex, genotyped batch, assessment center, Townsend deprivation index and the first 10 principal components of ancestry. Abbreviations: CI, confidence interval; OR, odds ratio; HR, hazard ratio; PRS, polygenic risk score; Ref, reference.

**Table S24.** RA risks according to genetic (PRS) and lifestyle categories after redefining smoking variable (Prospective analysis).

| **Subgroups** | | **All** | | **Men** | | **Women** | |
| --- | --- | --- | --- | --- | --- | --- | --- |
|  |  | **HR (95%CI)** | ***P*** | **HR (95%CI)** | ***P*** | **HR (95%CI)** | ***P*** |
| Low genetic risk | |  |  |  |  |  |  |
|  | Favorable | **Ref** |  | **Ref** |  | **Ref** |  |
|  | Intermediate | 0.878 (0.67, 1.151) | 1.95E-04 | 1.666 (1.127, 2.461) | 0.010 | 1.466 (1.116, 1.926) | 0.006 |
|  | Unfavorable | 1.225 (0.885, 1.696) | 8.63E-06 | 1.75 (1.043, 2.936) | 0.034 | 2.013 (1.427, 2.84) | 6.73E-05 |
|  |  | *P* _trend_ = 2.44E-07 |  | *P* _trend_ = 0.004 |  | *P* _trend_ = 1.04E-05 |  |
| Intermediate genetic risk | |  |  |  |  |  |  |
|  | Favorable | 0.903 (0.672, 1.214) | 0.003 | 1.512 (1.084, 2.11) | 0.015 | 1.245 (0.99, 1.565) | 0.061 |
|  | Intermediate | 1.111 (0.865, 1.429) | 5.77E-06 | 1.846 (1.315, 2.591) | 3.96E-04 | 1.701 (1.35, 2.143) | 6.45E-06 |
|  | Unfavorable | 1.769 (1.347, 2.322) | 8.96E-16 | 2.524 (1.709, 3.727) | 3.27E-06 | 2.57 (1.972, 3.349) | 2.82E-12 |
|  |  | *P* _trend_ = 3.35E-14 |  | *P* _trend_ = 4.53E-04 |  | *P* _trend_ = 4.29E-12 |  |
| High genetic risk | |  |  |  |  |  |  |
|  | Favorable | 1.243 (0.903, 1.711) | 6.90E-14 | 2.393 (1.697, 3.375) | 6.49E-07 | 1.979 (1.56, 2.511) | 1.87E-08 |
|  | Intermediate | 1.798 (1.395, 2.318) | 1.94E-22 | 2.989 (2.108, 4.236) | 7.75E-10 | 2.554 (2.004, 3.256) | 3.53E-14 |
|  | Unfavorable | 2.534 (1.908, 3.366) | 3.94E-33 | 4.315 (2.886, 6.451) | 1.04E-12 | 3.995 (3.017, 5.29) | 4.02E-22 |
|  |  | *P* _trend_ = 1.08E-11 |  | *P* _trend_ = 2.27E-04 |  | *P* _trend_ = 5.47E-09 |  |

Note: Defined by quintiles of PRS: low (the bottom quartile), intermediate (quintiles 2-3) and high (the top quintile). Participants were divided into favorable (3 healthy lifestyle factors), intermediate (2 healthy lifestyle factors), or unfavorable (0 or 1 healthy lifestyle factor) according to the number of lifestyle factors. *P* _trend_ is P values for Cochran-Armitage trend test. Associations were adjusted for age, sex, genotyped batch, assessment center, Townsend deprivation index and the first 10 principal components of ancestry. Abbreviations: CI, confidence interval; OR, odds ratio; HR, hazard ratio; PRS, polygenic risk score; Ref, reference.

**Table S25.** Additive interactions between genetic and lifestyle categories in test set (Case-control analysis).

| **Lifestyle** |  | **Genetic risk** | **RERI (95% CI)** | ***P*** | **AP (95% CI)** | ***P*** |
| --- | --- | --- | --- | --- | --- | --- |
| All |  |  |  |  |  |  |
| Intermediate | × | Intermediate risk | -0.058 (-0.405, 0.289) | 0.628 | -0.033 (-0.227, 0.162) | 0.371 |
| Intermediate | × | High risk | 0.710 (0.271, 1.148) | **7.58E-04** | 0.228 (0.089, 0.367) | **6.41E-04** |
| Unfavorable | × | Intermediate risk | 0.409 (-0.069, 0.886) | **0.047** | 0.133 (-0.023, 0.288) | **0.047** |
| Unfavorable | × | High risk | 1.614 (0.945, 2.283) | **1.12E-06** | 0.332 (0.215, 0.450) | **1.43E-08** |
| Men |  |  |  |  |  |  |
| Intermediate | × | Intermediate risk | -0.373 (-1.186, 0.441) | 0.815 | -0.169 (-0.519, 0.180) | 0.171 |
| Intermediate | × | High risk | 0.574 (-0.398, 1.546) | 0.124 | 0.148 (-0.102, 0.398) | 0.123 |
| Unfavorable | × | Intermediate risk | -0.318 (-1.459, 0.823) | 0.707 | -0.085 (-0.388, 0.218) | 0.290 |
| Unfavorable | × | High risk | 1.871 (0.355, 3.386) | **0.008** | 0.282 (0.085, 0.480) | **0.003** |
| Women |  |  |  |  |  |  |
| Intermediate | × | Intermediate risk | 0.049 (-0.332, 0.431) | 0.400 | 0.03 (-0.203, 0.263) | 0.401 |
| Intermediate | × | High risk | 0.774 (0.280, 1.268) | **1.06E-03** | 0.266 (0.100, 0.432) | **8.27E-04** |
| Unfavorable | × | Intermediate risk | 0.725 (0.187, 1.262) | **0.004** | 0.243 (0.066, 0.419) | **0.004** |
| Unfavorable | × | High risk | 1.616 (0.854, 2.379) | **1.62E-05** | 0.366 (0.222, 0.510) | **3.11E-07** |

Note: Defined by quintiles of PRS: low (the bottom quartile), intermediate (quintiles 2-3) and high (the top quintile). Participants were divided into favorable (3 healthy lifestyle factors), intermediate (2 healthy lifestyle factors), or unfavorable (0 or 1 healthy lifestyle factor) according to the number of lifestyle factors. Associations were adjusted for age, sex, genotyped batch, assessment center, Townsend deprivation index and the first 10 principal components of ancestry. Abbreviations: CI, confidence interval; RERI, relative excess risk for interaction; AP, attributable proportion due to interaction; Ref, reference.

**Table S26.** Additive interactions between genetic and lifestyle categories in test set (Prospective analysis).

| **Lifestyle** |  | **Genetic risk** | **RERI (95% CI)** | ***P*** | **AP (95% CI)** | ***P*** |
| --- | --- | --- | --- | --- | --- | --- |
| All |  |  |  |  |  |  |
| Intermediate | × | Intermediate risk | -0.035 (-0.392, 0.322) | 0.576 | -0.023 (-0.26, 0.214) | 0.424 |
| Intermediate | × | High risk | **0.544 (0.100, 0.987)** | **0.008** | **0.211 (0.039, 0.382)** | **0.008** |
| Unfavorable | × | Intermediate risk | 0.269 (-0.204, 0.741) | 0.133 | 0.110 (-0.085, 0.306) | 0.134 |
| Unfavorable | × | High risk | **0.868 (0.253, 1.483)** | **2.84E-03** | **0.246 (0.084, 0.408)** | **1.48E-03** |
| Men |  |  |  |  |  |  |
| Intermediate | × | Intermediate risk | -0.685 (-1.678, 0.308) | 0.912 | -0.350 (-0.802, 0.102) | 0.065 |
| Intermediate | × | High risk | 0.005 (-1.118, 1.128) | 0.496 | 0.002 (-0.331, 0.334) | 0.496 |
| Unfavorable | × | Intermediate risk | -0.203 (-1.363, 0.956) | 0.635 | -0.061 (-0.403, 0.282) | 0.364 |
| Unfavorable | × | High risk | 1.197 (-0.285, 2.679) | 0.057 | **0.219 (-0.033, 0.470)** | **0.044** |
| Women |  |  |  |  |  |  |
| Intermediate | × | Intermediate risk | 0.161 (-0.209, 0.530) | 0.197 | 0.118 (-0.161, 0.396) | 0.204 |
| Intermediate | × | High risk | **0.711 (0.237, 1.186)** | **0.002** | **0.304 (0.104, 0.503)** | **1.44E-03** |
| Unfavorable | × | Intermediate risk | 0.412 (-0.100, 0.925) | 0.057 | 0.191 (-0.047, 0.429) | 0.058 |
| Unfavorable | × | High risk | **0.731 (0.065, 1.397)** | **0.016** | **0.252 (0.038, 0.466)** | **0.011** |

Note: Defined by quintiles of PRS: low (the bottom quartile), intermediate (quintiles 2-3) and high (the top quintile). Participants were divided into favorable (3 healthy lifestyle factors), intermediate (2 healthy lifestyle factors), or unfavorable (0 or 1 healthy lifestyle factor) according to the number of lifestyle factors. Associations were adjusted for age, sex, genotyped batch, assessment center, Townsend deprivation index and the first 10 principal components of ancestry. Abbreviations: CI, confidence interval; RERI, relative excess risk for interaction; AP, attributable proportion due to interaction; Ref, reference.

**Table S27.** Lifestyle-by-single SNP interaction analysis on RA risk in test set.

| **Lifestyle** | **SNP** | **CHR** | **BP** | **Nearby genes** | **OR** | ***P*** |
| --- | --- | --- | --- | --- | --- | --- |
| Healthy lifestyle | rs10175798 | 2 | 30,449,594 | *H3P5, LBH* | 1.097 | 0.017 |
| Physical activity (Regular) | rs4452313 | 3 | 17,047,032 | *PLCL2* | 1.156 | 0.024 |
| Smoke (No) | rs9310852 | 3 | 27,784,997 | *EOMES, LINC01980* | 1.137 | 0.024 |
| Healthy lifestyle | rs111480654 | 4 | 27,782,743 | *-* | 1.494 | 0.006 |
| Physical activity (Regular) | rs111480654 | 4 | 27,782,743 | *-* | 1.778 | 0.017 |
| Smoke (No) | rs7731626 | 5 | 55,444,683 | *ANKRD55* | 0.891 | 0.048 |
| Diet (healthy) | rs144264189 | 6 | 26,311,416 | *-* | 0.521 | 0.002 |
| Physical activity (Regular) | rs72507814 | 6 | 29,541,566 | *-* | 1.398 | 0.010 |
| Smoke (No) | rs4713242 | 6 | 29,718,220 | *HCG9P5, IFITM4P* | 0.802 | 0.001 |
| Physical activity (Regular) | rs35590025 | 6 | 30,992,111 | *MUC21, LOC729792* | 0.795 | 0.038 |
| BMI (Moderate) | rs8133843 | 21 | 36,738,242 | *RUNX1* | 1.139 | 0.040 |

Note: Participants were divided into favorable (3 healthy lifestyle factors), intermediate (2 healthy lifestyle factors), or unfavorable (0 or 1 healthy lifestyle factor) according to the number of lifestyle factors. Abbreviations: SNP, single nucleotide polymorphism, CHR, chromosome; BP, physical position, OR, odds ratio.
